# Benzoimidazolium-derived dimeric and hydride n-dopants for organic electron-transport materials: impact of substitution on structures, electrochemistry, and reactivity

**DOI:** 10.3762/bjoc.19.121

**Published:** 2023-11-01

**Authors:** Swagat K Mohapatra, Khaled Al Kurdi, Samik Jhulki, Georgii Bogdanov, John Bacsa, Maxwell Conte, Tatiana V Timofeeva, Seth R Marder, Stephen Barlow

**Affiliations:** 1 Center for Organic Photonics and Electronics and School of Chemistry and Biochemistry, Georgia Institute of Technology, Atlanta, GA 80007, United States; 2 Department of Industrial and Engineering Chemistry, Institute of Chemical Technology—Indian Oil Campus, ITT Kharagpur Extension Center, Bhubaneswar 751013 Odisha, India; 3 Department of Chemistry, New Mexico Highlands University, Las Vegas, New Mexico 87701, United States; 4 Crystallography Lab, Emory University, Atlanta, Georgia 30322, United States; 5 Renewable and Sustainable Energy Institute (RASEI), University of Colorado Boulder, Boulder, Colorado 80309, United States; 6 Department of Chemical and Biological Engineering and Department of Chemistry, University of Colorado Boulder, Boulder, Colorado 80309, United States; 7 National Renewable Energy Laboratory, Chemistry and Nanoscience Center, Golden, Colorado, 80401, United States

**Keywords:** benzoimidazole, crystal structure, kinetics, n-dopant, reduction

## Abstract

1,3-Dimethyl-2,3-dihydrobenzo[*d*]imidazoles, **1H**, and 1,1',3,3'-tetramethyl-2,2',3,3'-tetrahydro-2,2'-bibenzo[*d*]imidazoles, **1_2_**, are of interest as n-dopants for organic electron-transport materials. Salts of 2-(4-(dimethylamino)phenyl)-4,7-dimethoxy-, 2-cyclohexyl-4,7-dimethoxy-, and 2-(5-(dimethylamino)thiophen-2-yl)benzo[*d*]imidazolium (**1g–i^+^**, respectively) have been synthesized and reduced with NaBH_4_ to **1gH**, **1hH**, and **1iH**, and with Na:Hg to **1g_2_** and **1h_2_**. Their electrochemistry and reactivity were compared to those derived from 2-(4-(dimethylamino)phenyl)- (**1b^+^**) and 2-cyclohexylbenzo[*d*]imidazolium (**1e^+^**) salts. *E*(**1^+^**/**1^•^**) values for 2-aryl species are less reducing than for 2-alkyl analogues, i.e., the radicals are stabilized more by aryl groups than the cations, while 4,7-dimethoxy substitution leads to more reducing *E*(**1^+^**/**1^•^**) values, as well as cathodic shifts in *E*(**1_2_^•+^**/**1_2_**) and *E*(**1H^•+^**/**1H**) values. Both the use of 3,4-dimethoxy and 2-aryl substituents accelerates the reaction of the **1H** species with PC_61_BM. Because 2-aryl groups stabilize radicals, **1b_2_** and **1g_2_** exhibit weaker bonds than **1e_2_** and **1h_2_** and thus react with 6,13-bis(triisopropylsilylethynyl)pentacene (**VII**) via a “cleavage-first” pathway, while **1e_2_** and **1h_2_** react only via “electron-transfer-first”. **1h_2_** exhibits the most cathodic *E*(**1_2_^•+^**/**1_2_**) value of the dimers considered here and, therefore, reacts more rapidly than any of the other dimers with **VII** via “electron-transfer-first”. Crystal structures show rather long central C–C bonds for **1b_2_** (1.5899(11) and 1.6194(8) Å) and **1h_2_** (1.6299(13) Å).

## Introduction

Electrical doping of organic semiconductors can play an important role in tuning the properties of organic semiconductors for a variety of applications [1–5]. The most straightforward n-dopants for doping electron-transporting materials are simple one-electron reductants; however, to be effective for a wide range of semiconductors, they must exhibit low ionization energies and thus air sensitivity. One approach to circumvent this issue is to identify systems where the electron-transfer process is coupled to other chemical reactions, increasing the kinetic stability of the dopant to air, and thus increasing its ease of storage and handling.

**Figure 1:**
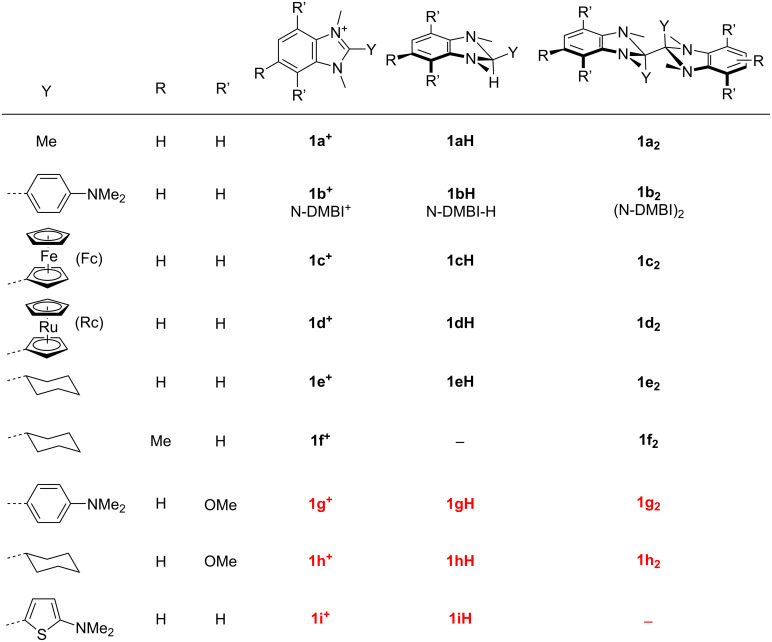
DMBI^+^, DMBI-H, and (DMBI)_2_ derivatives discussed in this work (new compounds in red).

Arguably, the most widely investigated air-inert n-dopants are 1,3-dimethyl-2,3-dihydrobenzo[*d*]imidazoles (DMBI-H, **1H**, Figure 1); these species have been known for decades (e.g., **1aH**, one of the simplest such derivatives, was first reported in 1954 [6]), but were only introduced in n-dopants in 2010, when Bao and co-workers reported the use of N-DMBI-H (**1bH**, Figure 1) to n-dope fullerenes [7]. Although widely used, due to their facile synthesis, structural tunability, and good air stability in the solid state, **1H** derivatives are relatively limited in dopant strength and their reactivity with organic semiconductors (SC) does not depend solely on the SC reduction potential, since the first step, at least in many cases, is a hydride transfer rather than an electron transfer [8–9]. Moreover, as well forming the desired semiconductor radical anion SC^•−^, and the stable DMBI^+^ (**1^+^**) species, a hydrogen atom must be lost from the dopant, in some cases resulting in the incorporation of hydrogen-reduced side products into the semiconductor film [9], although in other cases it may be lost as H_2_ [8,10–11].

The first report of a (DMBI)_2_ dimer (**1_2_**, Figure 1) was of **1a_2_** in 1984 [12]. More recently, dimers **1b_2_**–**1f_2_** (Figure 1) have been used as n-dopants [13–20]. They behave similarly to the closed-shell dimers formed by certain 19-electron transition-metal sandwich compounds [21–23], exhibiting moderate air stability and acting as quite strong dopants, reacting with semiconductors more rapidly and predictably than hydride donors such as the corresponding **1H** species [8], cleanly only to give SC^•–^ and the corresponding monomeric cations. However, **1_2_** dopants offer the possibility of more planar dopant ions than the organometallic dimers, which can be advantageous [19].

Although the impact of different 2-aryl Y groups on the reactivity of **1H** species have been examined [9,24], there has been no direct comparison of the solution reactivity (or doping behavior) of **1H** or **1_2_** reductants with Y = aryl substituents to that of their Y = alkyl counterparts, while there has also been limited effort on examining the effects of substituents on the benzimidazole 6-membered ring in either class of reductant [16,24]. Furthermore, there has been little work on Y = 2-thienyl **1H** derivatives. Here, we report two new dimers (**1g_2_** and **1h_2_**) and three new hydride donors (**1gH**, **1hH**, **1iH**). We also report crystal structures of several of these compounds and of several salts of the corresponding **1^+^** cations, and compare the electrochemistry and reactivity of these species.

## Results and Discussion

### Synthesis

Although an unsymmetrical **1_2_**-like molecule, 2-diethoxyphosphoryl-1,1',3,3'-tetramethyl-2,2',3,3'-tetrahydro-2,2'-bibenzo[*d*]imidazole, has been obtained from addition of HPO_3_Et_2_ across the central C=C bond of bis(1,3-dimethylbenzoimidazolinidin-2-ylidene) [25], **1_2_** dimers have generally been obtained by reductive electrochemical or chemical dimerization of **1^+^** cations [12–13,16,19,26]. **1H** derivatives can be obtained in a number of ways, including direct condensation of *N,N'*-dimethylphenylene-1,2-diamine derivatives with the appropriate aldehydes, YCHO [24,27], or borohydride reduction of **1^+^** salts [24]. The cations conversely can be obtained from **1H** derivatives, for example through hydride abstraction by Ph_3_C^+^ [13]. Alternatively, they can also be obtained by condensation of *N,N'*-dimethylphenylene-1,2-diamine derivatives with acid chlorides, YCOCl, or through the methylation of 2-substituted benzoimidazoles [24], which in turn can be obtained from condensation between phenylene-1,2-diamines and carboxylic acids YCO_2_H [28], oxidative condensation between YCHO and phenylene-1,2-diamines [29], or reductive condensation between YCHO and 2-nitroanilines [24].

**Scheme 1:**
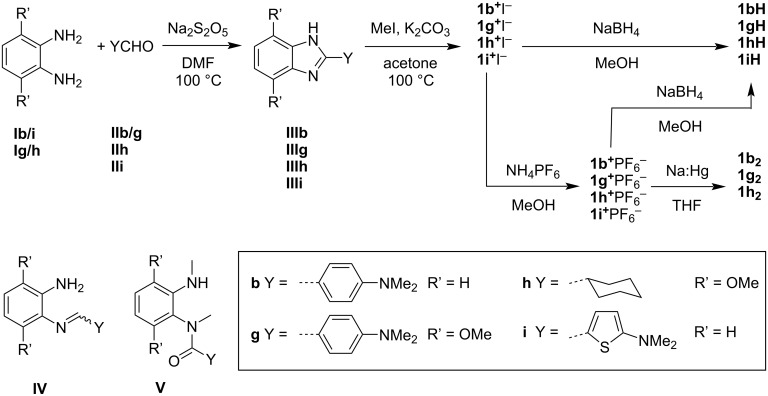
Synthesis of DMBI-H and (DMBI)_2_ derivatives and structures of side products.

In this work we condensed the appropriate YCHO aldehyde (**II**) and 1,2-diaminobenzene (**I**) derivatives in the presence of sodium metabisulfite (Na_2_S_2_O_5_) [29] to obtain the corresponding substituted benzimidazoles (**III**) in essentially quantitative yield (Scheme 1). In the absence of Na_2_S_2_O_5_, but under otherwise similar conditions, we obtained in some cases the imines in which one of the amino groups condenses with the aldehyde but where the subsequent second condensation and oxidation does not take place, i.e., structures of type **IV** (Scheme 1), which are known to be converted to benzimidazoles by various oxidants and/or catalysts [30–32]. The benzimidazoles were then doubly methylated with iodomethane (or methyl tosylate) to afford the benzimidazolium iodides (or tosylates), **1^+^**I^−^ (or **1^+^**OTs^−^), which were metathesized to the corresponding hexafluorophosphates, **1^+^**PF_6_^−^. Either I^−^ or PF_6_^−^ salt can then be converted to the corresponding **1H** derivative using NaBH_4_ in MeOH. The PF_6_^−^ salts are somewhat more soluble than the iodides in THF, so were reductively dimerized to **1_2_** in THF using Na:Hg, although reduction of **1i^+^**PF_6_^−^ failed to afford **1i_2_**. As we have noted before for other **1_2_** species, amides (**V**, Scheme 1) are encountered as both byproducts of dimer synthesis and dimer decomposition products [14]. **V** derivatives have also been obtained as pyrolysis products of a variety of Y = aryl **1H** derivatives [33], while **Vb** has also been found to be both a solution decomposition product of **1bH** [27,34] and a beneficial additive for a **1bH**-doped polymer [27], and has been crystallographically characterized [34]. In the case of molecules with aryl Y-substituents – **1b_2_** and **1g_2_** – the room-temperature ^1^H and ^13^C NMR spectra (see Supporting Information File 1, Figures S2, S26 and S27, and reference [26]) display more resonances than expected based on the highest symmetry possible for the molecule indicating that the sample represents neither solely a high-symmetry conformer, nor a mixture of rapidly exchanging lower symmetry conformers. In the case of **1b_2_** all the proton resonances are rather broad, and variable-temperature experiments (see Supporting Information File 1, Figure S2) showed further broadening and then coalescence of some of these peaks on increasing the temperature, consistent with the room-temperature spectrum being affected by restricted rotation; interestingly the crystal structure of **1b_2_** contains molecules with two very different conformations (see below).

The **1_2_** dimers are somewhat more sensitive to air than the corresponding **1H** hydrides, but are all sufficiently stable as solids that they can briefly be handled in air, for example, for weighing. The solids do decompose slowly in air, although we have not quantified this; in inert atmosphere, however, they are completely stable (at least 4 months for solid **1b_2_**). Both **1H** and **1_2_** derivatives decompose more rapidly on exposure to air in solution. In CDCl_3_ decomposition is rapid, consistent with the reactivity of many reductants with that solvent. In C_6_D_6_ these compounds are more stable, allowing, for example, rapid acquisition of a ^1^H NMR spectrum; however, handling under nitrogen is advisable as these species completely decompose to **V** (and perhaps **1^+^** species) on timescales of hours to days (see Supporting Information File 1, Figures S3–S5).

### Crystal structures

**Figure 2:**
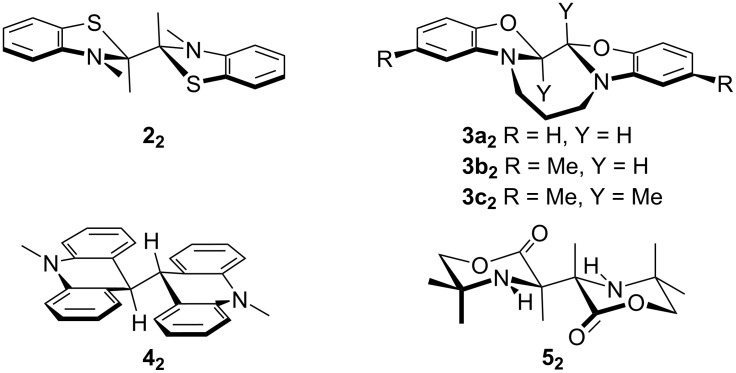
Crystallographically characterized molecules related to DMBI dimers.

**Figure 3:**
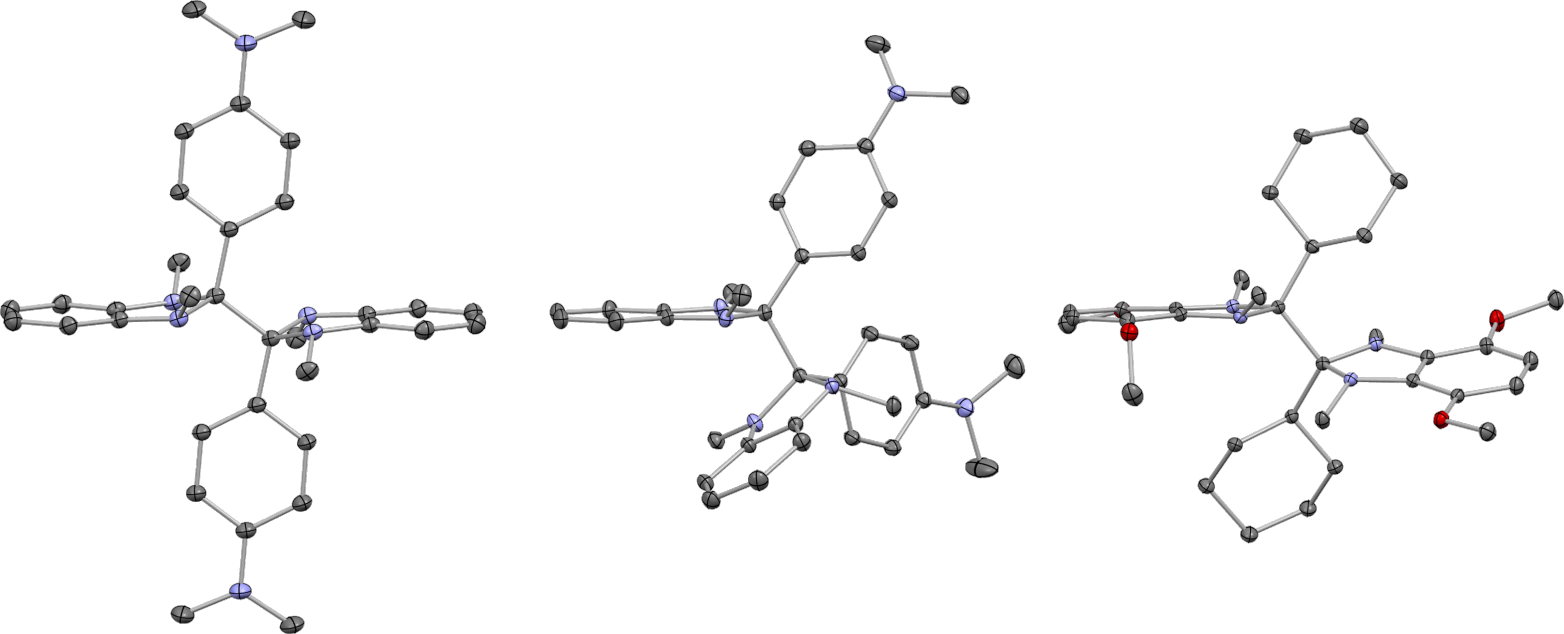
Molecular structures from the single crystal structures of **1b_2_** (two crystallographically inequivalent molecules, left and center) and **1h_2_** (right), shown with 50% thermal ellipsoids and excluding disorder in **1h_2_** and hydrogen atoms for clarity.

**Figure 4:**
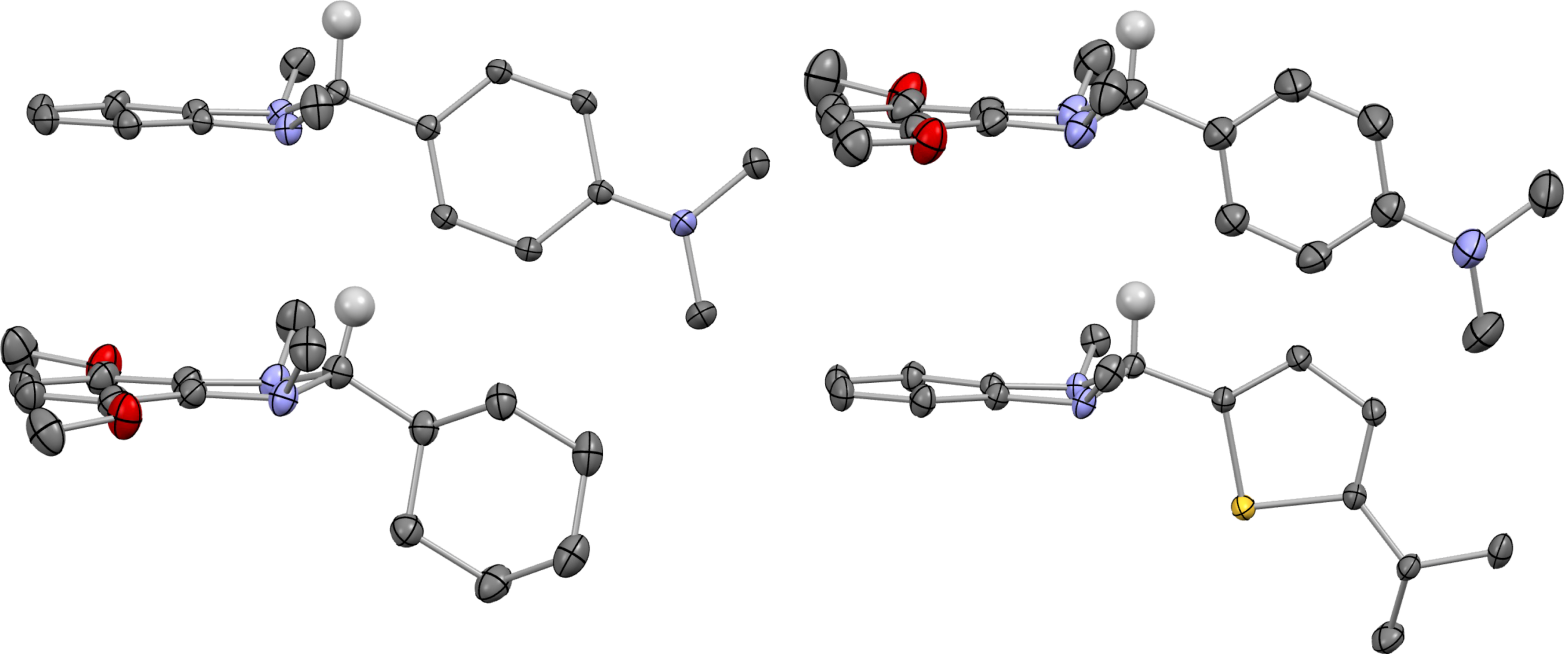
Molecular structures from the single crystal structures of **1bH** (upper left), **1gH** (upper right), **1hH** (lower left), and **1iH** (lower right), shown with 50% thermal ellipsoids and excluding hydrogen atoms for clarity, except for the hydridic 2-hydrogen atoms (located and refined for **1bH**, geometrically placed for the others).

We have determined the structures of two **1_2_** dimers, four **1H** derivatives (including **1bH**, the structure of which has previously been reported, but with somewhat lower precision than in the present work [34]), and three salts of **1^+^** cations using single-crystal X-ray diffraction. Here, we briefly discuss some of the more interesting structural findings; a more detailed comparison of structural parameters is given in the Supporting Information File 1, Table S2. In particular, we are aware of only two previously reported crystal structures of DMBI dimers [14], although several related structures of organic dimers, including those of benzothiazoline, benzoxazoline, acridanyl, morpholinonyl dimers (**2_2_–5_2_**, respectively, Figure 2) have been reported in different chemical contexts [35–38]. The crystal structure of (N-DMBI)_2_, **1b_2_** (Figure 3), contains two crystallographically inequivalent molecules that are geometrically rather different from each other. One of the molecules has crystallographic inversion (*C*_i_) symmetry, and approximate molecular *C*_2_*_h_* symmetry, and so has a perfectly staggered conformation around the central C–C bond and thus a Y–C–C–Y torsion angle of precisely 180°; the structure closely resembles those of the two inequivalent molecules in the structure of the previously reported Y = ferrocenyl derivative, **1c_2_** [14], or the molecule in the structure **2_2_** [35], all three of which also have *C*_i_ symmetry. The other conformer present, although also staggered, has no crystallographic, or even approximate molecular, symmetry and is characterized by a Y–C–C–Y torsion angle of 60.3°. The conformation found in the structure of the Y = cyclohexyl, R’ = OMe derivative, **1h_2_** (Figure 3), is somewhat similar to that previously reported for its non-methoxylated analogue **1e_2_** [14]; the **1h_2_** molecule does not have the crystallographic *C*_2_ symmetry of the latter, but does have approximate molecular *C*_2_ symmetry, while the Y–C–C–Y torsion angles for **1h_2_** and **1e_2_** are 149.4° and 140.3°, respectively, and thus both intermediate between the perfectly staggered (180° torsion) and neighboring eclipsed conformation (120°). The imidazole rings in the previously reported and present dimer structures are mostly somewhat folded towards a puckered envelope conformation, generally with the Y group in a pseudo-axial position and the 1,3-methyl groups and the central C–C bond in pseudo-equatorial positions, although for one of the monomers in the unsymmetrical conformer in the structure of (N-DMBI)_2_, **1b_2_,** the Y and central bond are pseudo-equatorial and pseudo-axial, respectively. However, this folding is generally much less pronounced than in **1H** derivatives (see below, Figure 4, and Table S2 in Supporting Information File 1) presumably since in the dimers both 2-substituents (Y and the other monomer unit) are fairly bulky, whereas in the hydrides there is a large difference in bulk between the hydridic H-atom and theY-group and thus a strong preference for Y to occupy a pseudo-equatorial position.

As with other **1_2_** species [14] and related organic [35,37–38] and organometallic dimers [22,39–46], the central C–C bond of the present dimers are rather long compared to typical C–C bonds, although not remarkably so given that these are hexasubstituted ethane derivatives. Values of 1.5899(11) and 1.6194(8) Å are found for the symmetrical and unsymmetrical conformers of **1b_2_**, respectively, while a value of 1.6299(13) Å is found for **1h_2_**; these may be compared to hexasubstituted central C–C bond length values of 1.595(5) and 1.601(5) Å for the two inequivalent molecules of the Y = Fc, R = R' = H derivative **1c_2_** [14], 1.640(4) Å for the Y = cyclohexyl, R = R' = H derivative **1e_2_** [14], 1.573 Å for **2_2_** [35], and 1.591 Å for **5_2_** [38], while (PhEt_2_C)_2_, a simple hexasubstituted ethane, exhibits a central C–C bond length of 1.635 Å [47]. The tetrasubstituted central C–C bond of **4_2_** is also rather long (1.58 Å) [37]. Bridged benzoxazoline dimers, **3_2_**, have, on the other hand, relatively short C–C central bonds, perhaps due to the influence of the propanediyl tether; the hexasubstituted bond of **3c_2_** is only 1.549(6) Å in length, while the tetrasubstituted bonds of **3a_2_** and **3b_2_** are even shorter [36].

The crystallographically determined central C–C bond lengths for **1b_2_** are shorter than that previously reported for the Y = cyclohexyl, R = R' = H derivative **1e_2_** (1.640(4) Å) [14], despite DFT calculations indicating that the former dimer is considerably more weakly bonded [8,14] and kinetic evidence for the “cleavage-first” mechanism occurring in doping reactions using **1b_2_** but not **1e_2_** (see below). We have previously noted a similar lack of correlation between bond length and bond dissociation energy in comparing the structures of **1c_2_** (Y = Fc; R = R' = H) and **1e_2_** (Y = cyclohexyl; R = R' = H) [14], and in comparing those of different organometallic dimers [22,46]. As noted in our previous work [14,22,46], the bond length depends on orbital overlap and steric strain in the dimer, whereas dissociation energetics also depend on the stability of the monomeric odd-electron species, which vary considerably; in the case of **1^•^** radicals an important factor is the ability of the Y substituent to delocalize spin density.

**Figure 5:**
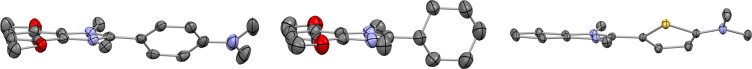
Structures of the cations from the single crystal structures of **1g^+^**I^−^ (left), **1h^+^**PF_6_^−^ (center), and **1i^+^**PF_6_^−^ (right), shown with 50% thermal ellipsoids and excluding hydrogen atoms and counter anions.

The **1H** structures (Figure 4) are similar to those of other DMBI-H structures in the literature [34,48–50] (and are compared in more detail in Supporting Information File 1, Table S2); in all cases the imidazole ring is folded in a “puckered envelope” conformation with the 2-Y and 1,3-dimethyl substituents in pseudo-equatorial positions and the reactive hydridic 2-H-atom pesudo-axial. The cation structures (Figure 5) give some insight into the variety of dopant-ion shapes and sizes that can be afforded by these types of dopants. The angle between the imidazolium ring and the aromatic ring of the **1g^+^**I^−^ is 41.5°, close to the range of values previously reported for **1b^+^** salts (42.5–52.5°) [19,34] and for salts of Y = Ph, R = R' = H cations with different counterions (42.0–54.9°) [51–53]. As expected, owing to reduced steric interactions associated with the five-membered rather than six-membered aromatic ring, the structure of **1i^+^**PF_6_^−^ contains a somewhat more planar cation (31.9°). Finally, we note that the new structures reported here mean that the **1b** and **1h** systems join the **1c** (Y = Fc; R = R' = H) system [50] as families for which **1^+^**, **1H**, and **1_2_** members are all crystallographically characterized.

### Electrochemistry

**Figure 6:**
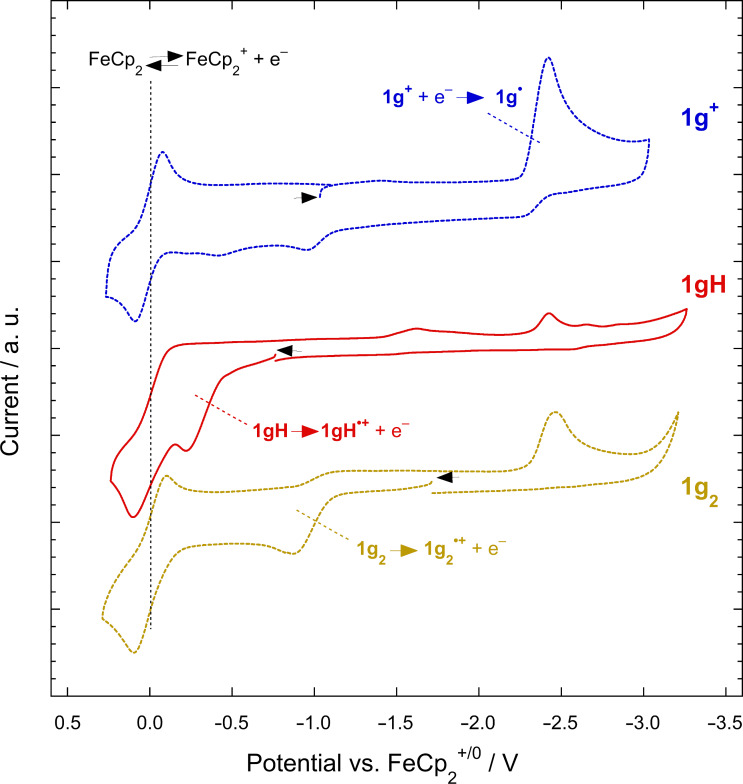
Cyclic voltammograms (50 mV s^−1^, THF, 0.1 M Bu_4_NPF_6_) of **1g^+^**PF_6_^–^, **1gH**, and **1g_2_**, in each case containing ferrocene as an internal reference. Black arrows indicate the starting point and scan initial direction for each voltammograms. Note that the oxidation peak of **1g_2_** is seen in the voltammogram of **1g^+^**PF_6_^–^ following scanning of the reduction peak, while the reduction peak of the cation is seen in the voltammograms of both **1gH** and **1g_2_** following scanning of the irreversible oxidation peaks.

The **1^+^**, **1H**, and **1_2_** species were investigated using cyclic voltammetry in THF/0.1 M Bu_4_NPF_6_ at a scan rate of 50 mV s^−1^. The voltammograms (shown for one series of compounds in Figure 6) were qualitatively similar to those reported and shown elsewhere for other compounds of the same classes [9,13,19,24], and the redox potentials are summarized in Table 1. The cations exhibit features assigned to *E*(**1^+^**/**1^•^**) that are non-reversible owing to the rapid dimerization of **1^•^**. These values are important in determining the overall thermodynamic reducing power of the dimers according to:


[1] $$E(1^{+}\text{/}0.51_{2})\;=E(1^{+}\text{/}1^{•})\;+\;\Delta G_{\text{diss}}(1_{2})/2F,$$

where Δ*G*_diss_(**1_2_**) is the free-energy change for dissociation of **1_2_** to **1^•^** (dissociation energetics are not estimated in the present work, but have been estimated using DFT calculations for **1b–e_2_** in previous works [8,14] and, in favorable cases, can be experimentally estimated using electron spin resonance [14] or using dissociation and dimerization barriers from reaction kinetics and variable scan-rate electrochemistry, respectively [54]) and where *F* is the Faraday constant. Similarly, at least for cases where the reactive hydrides of **1H** derivatives are ultimately lost as H_2_, the strength of **1H** dopants is given by:


[2] $$\begin{array}{c} E(1^{+},0.5{\text{H}}_{2}\text{/}1H)\;=E(1^{+}\text{/}1^{•})\;+\Delta G_{\text{diss}}(1H)/F\;\\ \;–\;\;\Delta G_{\text{diss}}\left({\text{H}}_{2}\right)/2F, \end{array}$$

where Δ*G*_diss_(**1H**) is the free-energy change for dissociation of **1H** to **1^•^** and H^•^ (again, not discussed in this work), and Δ*G*_diss_(H_2_) the free-energy change for dissociation of dihydrogen. The values of *E*(**1^+^**/**1^•^**) are also relevant to the kinetics of steps in doping reactions that involve **1^•^**, in particular for doping reactions in which the initial step is dimer dissociation and the second step is an electron transfer from **1^•^** to SC (or SC^•−^). The *E*(**1^+^**/**1^•^**) potentials for the Y = 4-dimethylaminophenyl **1b^+^/1b^•^** and **1g^+^/1g^•^** systems are both somewhat less reducing than those for their Y = cyclohexyl counterparts, **1e^+^/1e^•^** and **1h^+^/1h^•^,** respectively. These differences are also similar to those previously seen in the comparison of Y = metallocenyl systems **1c^+^/1c^•^** and **1d^+^/1d^•^** with **1e^+^/1e^•^** (and in the DFT-calculated ionization energies of **1c**–**e^•^**) [14,50] and are perhaps surprising since 4-(dimethylamino)phenyl and metallocenyl groups are π-donors, unlike cyclohexyl, and thus might be expected to be better able to stabilize an adjacent cation. However, aryl and metallocenyl substituents also stabilize adjacent radicals more effectively than alkyl groups and this effect is presumably dominant in the present case. The importance of radical stabilization may in part be because the positive charges in Y = H or alkyl **1^+^** ions is already substantially stabilized by the aromaticity of the benzimidazolium ions, whereas the spin densities of the corresponding **1^•^** radicals are highly localized; indeed DFT calculations for the Y = alkyl **1e^•^** derivative indicate spin density almost entirely on the 2-position of the five-membered ring, while for Y = aryl and metallocenyl examples **1b^•^**, **1c^•^**, and **1d^•^** there is substantial spin delocalization onto the Y-substituents [14,55]. Different extents of deviation from planarity in cations and radicals, as well as inductive effects, may also play a role.

**Table 1:** Electrochemical potentials (V) for DMBI derivatives^a^.

	*E*_red_(**1^+^**/**1^•^**)	*E*_ox_(**1H^•+^**/**1H**)	*E*_ox_(**1_2_^•+^**/**1_2_**)

**1b** (Y = C_6_H_4_-4-NMe_2_; R = R' = H)	−2.38^b^	−0.13^c^	−0.75^b^
**1c** (Y = Fc; R = R' = H)	−2.24^d^	−0.06^e^	−0.89^d^
**1d** (Y = Rc; R = R' = H)	−2.29^d^	−0.07^e^	−0.59^d^
**1e** (Y = cy-C_6_H_11_; R = R' = H)	−2.45^d^	−0.06	−0.64^d^
**1g** (Y = C_6_H_4_-4-NMe_2_; R = H; R' = OMe)	−2.42	−0.22	−0.87
**1h** (Y = cy-C_6_H_11_; R = H; R' = OMe)	−2.56	−0.11	−0.92
**1i** (Y = 2-C_4_H_3_S-5-NMe_2_; R = R' = H)	–2.05	−0.22	–

^a^vs FeCp_2_^+/0^ in THF, 0.1 M Bu_4_NPF_6_; ^b^data from reference [19]; ^c^data from reference [8]; ^d^data from reference [14]; ^e^data from reference [50].

The **1i^+^**/**1i^•^** (Y = 5-(dimethylamino)-2-thienyl; R = R' = H) potential is less reducing than that of **1b^+^**/**1b^•^** (Y = 4-dimethylaminophenyl; R = R' = H). 5-(Dimethylamino)-2-thienyl is more strongly π-donating than 4-dimethylaminophenyl, at least according to NMR and DFT data for molecules in which the (hetero)aryl group is more or less coplanar with a π-acceptor [56], although some tabulated Swain–Lupton substituent constants do suggest phenyl can be a stronger π-donor than thienyl towards another aryl ring [57]. Presumably inductive effects destabilizing **1i^+^**, different extents of planarization, and improved radical stabilization by the 5-(dimethylamino)-2-thienyl susbtituent play a role. As expected, R' = OMe groups on the six-membered benzimidazolium ring *do* have a net cation-stabilizing effect, resulting in **1g^•^** and **1h^•^** being more reducing monomers than their non-methoxylated analogues **1b^•^** and **1e^•^**, respectively.

Cyclic voltammograms of both **1H** and **1_2_** both reveal irreversible oxidations (with the corresponding **1^+^** reductions seen in subsequent reductive cycles, see Figure 6 for examples). These **1H^•+^**/**1H** and **1_2_^•+^**/**1_2_** potentials are relevant to the air stability of the hydrides and dimers, respectively, as well as to other processes in which **1H** or **1_2_** acts as an electron donor, such as the initation step proposed for the radical-chain dehalogenation of α-dihaloketones by a **1H** derivative [58] and dimer n-doping reactions that proceed via the “ET-first” mechanism (see below). In all cases the dimers are more easily oxidized, consistent with their greater air sensitivity. The impact of the Y-substituents on both **1H^•+^**/**1H** and **1_2_^•+^**/**1_2_** potentials is not straightforward; one would expect π-conjugated substituents to make little contribution to the HOMO of either **1H** or **1_2_** (as shown in calculated molecular orbitals for several examples [14,50,55,59–60]) and so the dependence of these potentials on Y is likely to be due to a combination of inductive effects and perhaps steric effects on the molecular conformation. As expected, methoxy R' substituents lead to **1H^•+^**/**1H** and **1_2_^•+^**/**1_2_** potentials that are more reducing than those for analogous species without these groups. **1h_2_** (Y = cyclohexyl, R = H, R' = MeO) is the most easily oxidized DMBI dimer that we have examined to date; however, it is a little less easily oxidized than [RuCp*(1,3,5-Me_3_C_6_H_3_)]_2_ (−1.09 V) [61] and, like [RuCp*(1,3,5-Me_3_C_6_H_3_)]_2_, can still be handled in air.

### Reactivity

**Figure 7:**
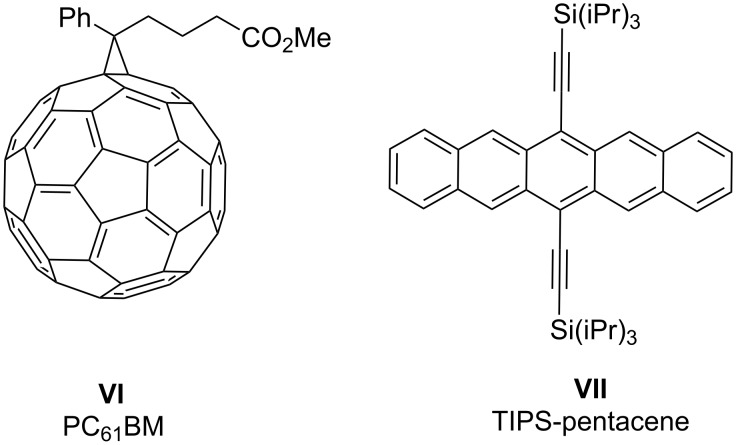
Acceptors used to examine reactivity of DMBI-H and (DMBI)_2_ derivatives.

**Figure 8:**
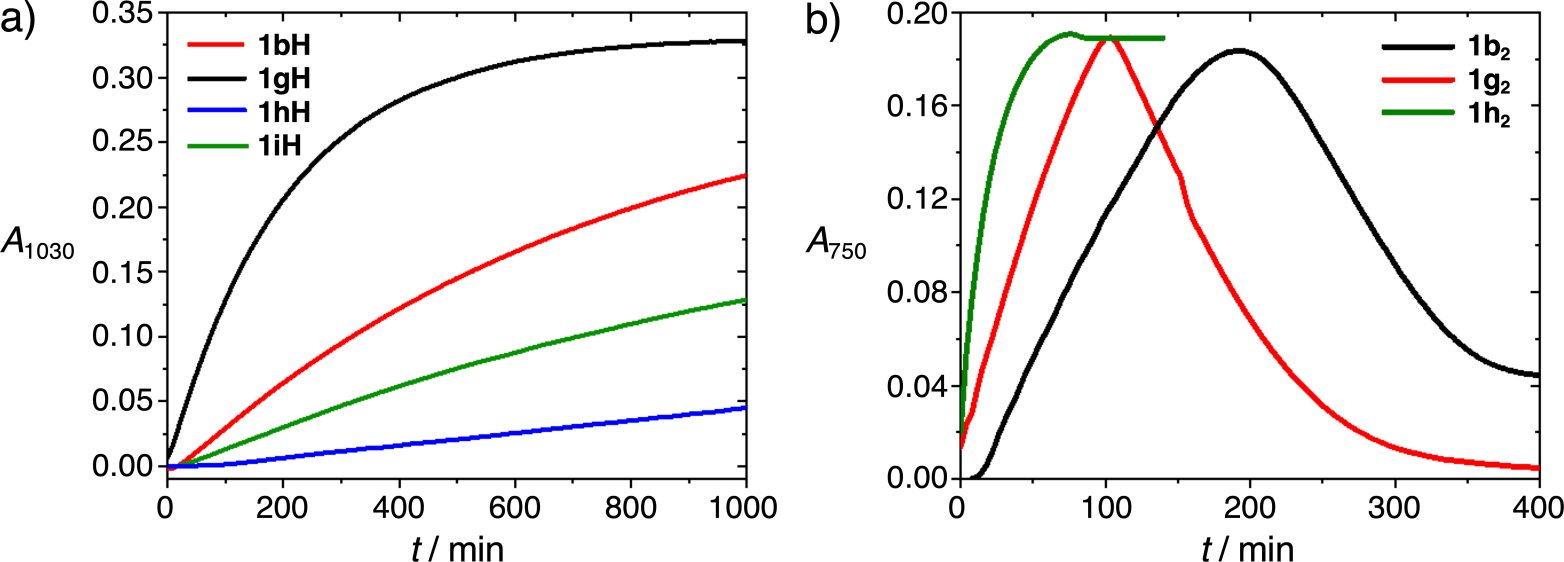
a) Temporal evolution of the absorbance at 1030 nm, corresponding to an absorption maximum of **VI^•–^**, when PC_61_BM (**VI**, 2.7 mM) is reacted with different **1H** derivatives (0.4 mM) in chlorobenzene at room temperature. b) Temporal evolution of the absorbance at 750 nm, corresponding to one of the absorption maxima of **VII^•–^**, when TIPS-pentacene (**VII**, 0.026 mM) is reacted with different **1_2_** derivatives (0.37 mM) in chlorobenzene at room temperature.

To compare the reactivity of the new compounds towards relevant organic semiconductors (SC), we have examined the reactions of the **1H** derivatives with the solubilized fullerene PC_61_BM (**VI**, Figure 7) and that of the **1_2_** derivatives with 6,13-bis(triisopropylsilylethynyl)pentacene (TIPS-pentacene, **VII**, Figure 7), since we have previously found that these dopant class/SC combinations often react on a timescale suitable for monitoring using UV–vis–NIR spectroscopy (**1H** derivatives do not react significantly with **VII** in solution at room temperature, while the reactions of **1_2_** derivatives and **VI** are very rapid) [9,14,50,61]. Figure 8a compares the evolution of the absorbance at 1030 nm, corresponding to a **VI^•–^** absorption maximum, when doping excess **VI** with **1H** derivatives in chlorobenzene at 293 K in the absence of light, air, and water. In each case the reaction is apparently first order in dopant, consistent with the rate law:


[3] $$d\left[VI^{•–}\right]/dt=k\left[1H\right]\left[VI\right]$$

previously demonstrated for **1bH** and **VI** [9]. The rate constants, *k*, obtained assuming this rate law are shown in Table 2 (the value for **3b** being similar to that previously determined [9]). One can anticipate, extending the Hammond postulate, that increased driving forces should correlate with reduced barriers and increased rate constants. Values of *k* do *not* correlate with the **1H^•^**^+^/**1H** potentials, but, at least when comparing aryl and alkyl Y substituents and when comparing R' = H and R' = OMe examples, do correlate with the expected stability of the resultant **1^+^** cations, which is also expected to correlate with the hydride donor strength of **1H**. This is consistent with previous findings that the first and rate-determining step of several **1H**/SC reactions, including **1H**/**VI** reactions, is *not* an electron transfer, but a hydride transfer [8–9]. There is conflicting evidence in the literature regarding the π-donor characteristics of phenyl and thienyl groups [56–57], while thienyl is more inductively electron-withdrawing [57], as noted in the electrochemical section; however, the observed rate constants for **1bH** and **1iH** suggest that 5-dimethylamino-2-thienyl affords less net charge stabilization than 4-dimethylaminophenyl.

**Table 2:** Rate constants for the reaction of DMBI-H derivatives and PC_61_BM.

	*k* [M^−1^ min^−1^]

**1bH** (Y = C_6_H_4_-4-NMe_2_; R = R' = H)	0.26
**1gH** (Y = C_6_H_4_-4-NMe_2_; R = H; R' = OMe)	0.48
**1hH** (Y = cy-C_6_H_11_; R = H; R' = OMe)	0.04
**1iH** (Y = 2-C_4_H_3_S-5-NMe_2_; R = R' = H)	0.13

Two reaction pathways have been established for the oxidation of organometallic and organic dimers. A “cleavage-first” mechanism, whereby the dimer is in equilibrium with a small concentration of the corresponding odd-electron monomer, which can then rapidly react with an acceptor such as an organic semiconductor (SC) through an exergonic electron transfer (ET), has been observed for the reactions of several relatively weakly bonded dimeric dopants (the Y = metallocenyl DMBI dimers **1c_2_** and **1d_2_** as well as various organometallic dimers) with **VII** [14,46,61], as well as in the oxidation of bis(3,5,5-trimethyl-2-morpholinon-3-yl), **5_2_** (Figure 2), by isatin derivatives [62]. In the alternative “ET-first” mechanism the first step is an endergonic dimer-to-SC ET; subsequent rapid cleavage of the odd-electron dimer cation affords the stable monomer cation and an odd-electron monomer, the latter then undergoing an exergonic ET to another SC molecule. The “ET-first” mechanism occurs in parallel with the “cleavage-first” mechanism for many of the **VII** doping reactions mentioned above and is the only mechanism seen for dimeric dopants that are more strongly bound (**1e_2_**, as well as various organometallic dimers including [RuCp*(1,3,5-Me_3_C_6_H_3_)]_2_) [14,46,61], as well as being observed in different contexts in, for example, the oxidation of **4_2_** by various quinone derivatives [63]. For both mechanisms, the first steps are typically rate determining and thus, in general, the rate law is:


[4] $$d\left[{\text{SC}}^{•–}\right]/dt=\;2k_{1}\left[1_{2}\right]\;+\;2k_{2}\left[1_{2}\right]\left[\text{SC}\right]\;,$$

where *k*_1_ and *k*_2_ are rate constants for the first steps of the “cleavage first” and “ET-first” pathways respectively, *k*_1_ being negligible in the case of strongly bound dimers.

Figure 8b compares the evolution of one of the distinctive **VII^ •–^** absorptions when doping **VII** with excess **1_2_** derivatives in chlorobenzene at 293 K in the absence of light, air, and water. In the case of the Y = 4-dimethylaminophenyl dimers **1b_2_** and **1g_2_**, the **VII^•–^** absorption grows in and then falls approximately linearly at a comparable rate. This type of plot is a signature of dimer/**VII** combinations for which the “cleavage-first” pathway is important and has previously been seen for the reactions of **VII** with **1c_2_**, **1d_2_**, (RhCp*Cp)_2_, and one of the isomers of [RuCp*{1,4-(Me_2_N)_2_C_6_H_4_}]_2_, all of which are calculated to be relatively weakly bonded [14,46,61]. Specifically, this behavior is consistent with a “cleavage-first” mechanism in which the initial cleavage is rate determining and for which the resultant one-electron monomers are capable of reducing *both* **VII** to **VII^•–^** (−1.55 V) *and* **VII^•–^** to **VII^2–^** (−1.93 V); since the cleavage is rate determining, **VII** will be converted to **VII^•–^** and then, when excess dimer is used, to **VII^2–^** with a comparable rate constant. Indeed spectra obtained at long-reaction times (see Supporting Information File 1, Figure S7) are similar to those previously attributed to **VII^2–^** [14,46,61], such as the reaction product of **VII** and Na:K. On the other hand, when *only* the “ET-first” mechanism is operative, the conversion of **VII^•–^** to **VII^2–^** will be much slower, if it is even observable, than the initial formation of **VII^•–^** from **VII** due to the considerably greater endergonicity expected for this step. This is seen for the solution reaction of **1h_2_**, where, as in the case of non-methoxylated analogue **1e_2_**, only the formation of **VII^•–^** is seen and the growth in its absorbance can be fitted as first order in **VII**. Returning to the case of **1b_2_** and **1g_2_**, we note that the rise in **VII^•–^** absorption is neither zero-order nor first-order in **VII**, consistent with *both* mechanisms contributing, as previously demonstrated by more extensive investigations in the case of **1c_2_**, **1d_2_**, and (RhCp*Cp)_2_ [14,61]. Thus, the Y = alkyl derivative (**1h_2_**, “ET-first” only) appears to be more strongly bonded than its Y = aryl counterparts (**1b_2_**, **1g_2_**, both mechanisms), consistent with previous DFT calculations for **1b_2_** and **1e_2_** (Δ*U*_diss_ = 163 and 210 kJ mol^–1^, respectively) and with the expected impact of the different Y substituents on monomer radical stability. In addition, the reaction of **1h_2_** and **VII** to form **VII^•–^** under the conditions used in the present study is complete much sooner than reactions using **1b_2_** or **1g_2_**, consistent with the ET-first reaction of **3h_2_** being more rapid than that for either **1b_2_** or **1g_2_**. Furthermore, the presumed “cleavage-first” reductions of **VII^•–^** to **VII^2–^** proceed only slightly faster for **1g_2_** than for its non-methoxylated analogue **1b_2_**, suggesting the OMe groups only slightly weaken the bond in the latter and that the difference in the rates of formation of **VII^•–^** with these two dimers is largely due to differences in the rate of the **1_2_**-to-**VII** ET reaction. Furthermore, the ordering of ET-first rates (**1h_2_** > **1g_2_** > **1b_2_** > **1e_2_**, that for **1e_2_** being estimated by extrapolating previously reported parameters to the present conditions of temperature and concentration) reflecting the trend expected based on the *E*(**1_2_^•+^**/**1_2_**) values of Table 1.

It is worth noting that, although we see evidence for the “cleavage-first” mechanism in the reactions of **1b_2_** and **1g_2_** with **VII** at these specific concentrations, the “ET-first” mechanism will dominate these reactions (as well as those of the same dopants with more readily reduced SCs) under typical doping conditions, where SC and sub-stoichiometric dimer are mixed in solution prior to spin-coating at much higher concentrations. However, as we have previously noted, there are potential advantages and disadvantages for dimers for which the cleavage-first pathway is viable and those for which it is not. For the former class, doping in solution will proceed as long as *E*(SC/SC^•–^) is less reducing than *E*(**1^+^**/0.5**1_2_**), whereas in the latter this limit can only be reached as long as the **1_2_**-to-SC ET step is kinetically feasible under the reaction conditions. Moreover, for a given monomer redox potential, *E*(**1^+^**/**1^•^**), a weakly bound dimer will be thermodynamically stronger (Equation 1) although, in some cases the effects of structural change on *E*(**1^+^**/**1^•^**) and Δ*G*_diss_(**1_2_**) partially cancel one another, as in the comparison of **1b_2_** vs **1e_2_** or **1g_2_** vs **1h_2_** (i.e., for Y = 4-dimethylaminophenyl, dimers are more weakly bound and monomers less reducing that for Y = cyclohexyl). Conversely, the combination of a strongly bound dimer and an acceptor with *E*(SC/SC^•−^) with the reach of *E*(**1^+^**/0.5**1_2_**), but sufficiently cathodic that ET is very slow, could permit activation of doping by an external stimulus, such as photoexcitation, when desired, for example subsequent to processing.

## Conclusion

In conclusion we have reported a number of new DMBI-H and (DMBI)_2_ reductants and compared their structures, electrochemistry, and reactivity with those of previously reported analogues. The structures show similar features to other related compounds, notably the dimers show long central C–C bonds. The *E*(**1^+^**/**1^•^**) potentials depend strongly on the 2-substituents (Y), become increasing reducing (more negative) in the order Y = 5-(dimethylamino)thiophen-2-yl < 4-(dimethylamino)phenyl < cyclohexyl, indicating the effects of radical stabilization are more important than those of cation stabilization, while the *E*(**1H^•+^**/**1H**) and *E*(**1_2_^•+^**/**1_2_**) potentials are less strongly and clearly affected by the 2-substituents. On the other hand, methoxy R’ substituents lead to more reducing values of *E*(**1^+^**/**1^•^**), *E*(**1H^•+^**/**1H**), and *E*(**1_2_^•+^**/**1_2_**) than for R’ = H analogues. The reaction rates of **1H** with PC_61_BM (**VI**) increase in the order Y = cyclohexyl < 5-(dimethylamino)thiophen-2-yl < 4-(dimethylamino)phenyl and R’ = H < MeO, broadly consistent with the anticipated influence of these substituents on the DMBI^+^ stability, as expected for a hydride-transfer reaction. The rates of reactions of the dimers with TIPS-pentacene (**VII**) follow a more complex pattern: examples with Y = cyclohexyl react solely via an “electron-transfer-first” mechanism, consistent with a relatively strongly bonded dimer, whereas Y = 4-(dimethylamino)phenyl derivatives also react by a “cleavage-first” mechanism, consistent with a weaker central bond, which in turn is consistent with stabilization of the monomeric radicals by the 2-aryl substituents. The Y = cyclohexyl, R’ = OMe dimer reacts most rapidly with TIPS-pentacene via the “ET-first” mechanism, consistent with this dimer also exhibiting the most cathodic value of *E*(**1_2_^•+^**/**1_2_**). Overall, this study gives insight into how substituents have different effects on the reactivity of DMBI-H derivatives and of (DMBI)_2_ species, and may help provide guidance for dopant selection and for future dopant design.

## Supporting Information

File 1 Synthetic and other experimental procedures, details of crystal-structure determinations, variable-temperature NMR data, stability data, optical spectra for reactivity studies, and NMR spectra of new compounds.

## References

[1] Walzer K, Maennig B, Pfeiffer M, Leo K (2007). Chem Rev.

[2] Russ B, Glaudell A, Urban J J, Chabinyc M L, Segalman R A (2016). Nat Rev Mater.

[3] Lüssem B, Keum C-M, Kasemann D, Naab B, Bao Z, Leo K (2016). Chem Rev.

[4] Wang Z-K, Liao L-S (2018). Adv Opt Mater.

[5] Barlow S, Marder S R, Lin X, Zhang F, Kahn A, Skotheim T A, Reynolds J, Thompson B C (2019). Electrial Doping of Organic Semiconductors with Molecular Oxidants and Reductants. Conjugated Polymers.

[6] Wahl H (1954). Bull Soc Chim Fr.

[7] Wei P, Oh J H, Dong G, Bao Z (2010). J Am Chem Soc.

[8] Jhulki S, Un H-I, Ding Y-F, Risko C, Mohapatra S K, Pei J, Barlow S, Marder S R (2021). Chem.

[9] Naab B D, Guo S, Olthof S, Evans E G B, Wei P, Millhauser G L, Kahn A, Barlow S, Marder S R, Bao Z (2013). J Am Chem Soc.

[10] Guo H, Yang C-Y, Zhang X, Motta A, Feng K, Xia Y, Shi Y, Wu Z, Yang K, Chen J (2021). Nature.

[11] Pallini F, Mattiello S, Manfredi N, Mecca S, Fedorov A, Sassi M, Al Kurdi K, Ding Y-F, Pan C-K, Pei J (2023). J Mater Chem A.

[12] Ludvík J, Pragst F, Volke J (1984). J Electroanal Chem Interfacial Electrochem.

[13] Naab B D, Zhang S, Vandewal K, Salleo A, Barlow S, Marder S R, Bao Z (2014). Adv Mater (Weinheim, Ger).

[14] Zhang S, Naab B D, Jucov E V, Parkin S, Evans E G B, Millhauser G L, Timofeeva T V, Risko C, Brédas J-L, Bao Z (2015). Chem – Eur J.

[15] Naab B D, Gu X, Kurosawa T, To J W F, Salleo A, Bao Z (2016). Adv Electron Mater.

[16] Yuan D, Huang D, Zhang C, Zou Y, Di C-a, Zhu X, Zhu D (2017). ACS Appl Mater Interfaces.

[17] Schwarze M, Gaul C, Scholz R, Bussolotti F, Hofacker A, Schellhammer K S, Nell B, Naab B D, Bao Z, Spoltore D (2019). Nat Mater.

[18] Al Kurdi K, Gregory S A, Jhulki S, Conte M, Barlow S, Yee S K, Marder S R (2020). Mater Adv.

[19] Un H-I, Gregory S A, Mohapatra S K, Xiong M, Longhi E, Lu Y, Rigin S, Jhulki S, Yang C-Y, Timofeeva T V (2019). Adv Energy Mater.

[20] Lungwitz D, Joy S, Mansour A E, Opitz A, Karunasena C, Li H, Panjwani N A, Moudgil K, Tang K, Behrends J (2023). J Phys Chem Lett.

[21] Guo S, Kim S B, Mohapatra S K, Qi Y, Sajoto T, Kahn A, Marder S R, Barlow S (2012). Adv Mater (Weinheim, Ger).

[22] Mohapatra S K, Fonari A, Risko C, Yesudas K, Moudgil K, Delcamp J H, Timofeeva T V, Brédas J-L, Marder S R, Barlow S (2014). Chem – Eur J.

[23] Mohapatra S K, Marder S R, Barlow S (2022). Acc Chem Res.

[24] Zhu X-Q, Zhang M-T, Yu A, Wang C-H, Cheng J-P (2008). J Am Chem Soc.

[25] Küçükbay H, Çetinkaya E, Çetinkaya B, Lappert M F (1997). Synth Commun.

[26] Pham P H, Barlow S, Marder S R, Luca O R (2023). Chem Catal.

[27] Pallini F, Mattiello S, Cassinelli M, Rossi P, Mecca S, Tan W L, Sassi M, Lanzani G, McNeill C R, Caironi M (2022). ACS Appl Energy Mater.

[28] Lim C-H, Ilic S, Alherz A, Worrell B T, Bacon S S, Hynes J T, Glusac K D, Musgrave C B (2019). J Am Chem Soc.

[29] Ghosh R, Kushwaha A, Das D (2017). J Phys Chem B.

[30] Crippa G B, Maffei S (1941). Gazz Chim Ital.

[31] Balachandran K S, George M V (1973). Indian J Chem.

[32] Speier G, Párkányi L (1986). J Org Chem.

[33] Reddy A P R, Veeranagaiah V, Ratnam C V (1985). Indian J Chem.

[34] Bardagot O, Aumaître C, Monmagnon A, Pécaut J, Bayle P-A, Demadrille R (2021). Appl Phys Lett.

[35] Miler-Srenger E (1973). Acta Crystallogr, Sect B: Struct Crystallogr Cryst Chem.

[36] Ramirez-Montes P I, Ochoa M E, Rodríguez V, Santillan R, García-Ortega H, Rodríguez P, Farfán N (2012). Tetrahedron Lett.

[37] Preuss J, Zanker V, Gieren A (1977). Acta Crystallogr, Sect B: Struct Crystallogr Cryst Chem.

[38] Haltiwanger R C, Koch T H, Olesen J A, Kim C S, Kim N K (1977). J Am Chem Soc.

[39] Andrianov V G, Struchkov Y T, Petrakova V A, Vol'kenau N A (1986). Koord Khim.

[40] Gaudet M V, Hanson A W, White P S, Zaworotko M J (1989). Organometallics.

[41] Lee S, Lovelace S R, Arford D J, Geib S J, Weber S G, Cooper N J (1996). J Am Chem Soc.

[42] Hsu S C N, Yeh W-Y, Lee G-H, Peng S-M (1998). J Am Chem Soc.

[43] Hitchcock P B, Lappert M F, Protchenko A V (2001). J Am Chem Soc.

[44] Shao L, Geib S J, Badger P D, Cooper N J (2002). J Am Chem Soc.

[45] Tamm M, Bannenberg T, Fröhlich R, Grimme S, Gerenkamp M (2004). Dalton Trans.

[46] Longhi E, Risko C, Bacsa J, Khrustalev V, Rigin S, Moudgil K, Timofeeva T V, Marder S R, Barlow S (2021). Dalton Trans.

[47] Kratt G, Beckhaus H-D, Lindner H J, Rüchardt C (1983). Chem Ber.

[48] Beauchamp A L, Montgrain F, Wuest J D (1987). Acta Crystallogr, Sect C: Cryst Struct Commun.

[49] Mas-Marzá E, Poyatos M, Sanaú M, Peris E (2004). Inorg Chem.

[50] Zhang S, Moudgil K, Jucov E, Risko C, Timofeeva T V, Marder S R, Barlow S (2019). Inorg Chim Acta.

[51] Wright A G, Weissbach T, Holdcroft S (2016). Angew Chem, Int Ed.

[52] Mehrotra S, Raje S, Jain A K, Angamuthu R (2017). ACS Sustainable Chem Eng.

[53] Li X, Hao P, Shen J, Fu Y (2018). Dalton Trans.

[54] Moudgil K, Mann M A, Risko C, Bottomley L A, Marder S R, Barlow S (2015). Organometallics.

[55] Uebe M, Yoshihashi Y, Noda K, Matsubara M, Ito A (2018). J Mater Chem C.

[56] Kwon O, Barlow S, Odom S A, Beverina L, Thompson N J, Zojer E, Brédas J-L, Marder S R (2005). J Phys Chem A.

[57] Hansch C, Leo A, Taft R W (1991). Chem Rev.

[58] Tanner D D, Chen J J (1989). J Org Chem.

[59] Riera-Galindo S, Orbelli Biroli A, Forni A, Puttisong Y, Tessore F, Pizzotti M, Pavlopoulou E, Solano E, Wang S, Wang G (2019). ACS Appl Mater Interfaces.

[60] Zeng Y, Zheng W, Guo Y, Han G, Yi Y (2020). J Mater Chem A.

[61] Guo S, Mohapatra S K, Romanov A, Timofeeva T V, Hardcastle K I, Yesudas K, Risko C, Brédas J-L, Marder S R, Barlow S (2012). Chem – Eur J.

[62] Bennett R W, Wharry D L, Koch T H (1980). J Am Chem Soc.

[63] Colter A K, Lai C C, Parsons A G, Ramsey N B, Saito G (1985). Can J Chem.

[64] Al Kurdi K (2021). Investigating Charge Transport in Conjugated Organic Materials.

