# Supplementary material for: Benzoimidazolium-derived dimeric and hydride n-dopants for organic electron-transport materials: impact of substitution on structures, electrochemistry, and reactivity
Source: Beilstein J Org Chem. 2023 Nov 1;19:1651–63. doi: 10.3762/bjoc.19.121 (PMC10630679; doi:10.3762/bjoc.19.121)
Supplement: File 1 — Synthetic and other experimental procedures, details of crystal-structure determinations, variable-temperature NMR data, stability data, optical spectra for reactivity studies, and NMR spectra of new compounds. [file Beilstein_J_Org_Chem-19-1651-s001.pdf]

BEILSTEIN JOURNAL OF ORGANIC CHEMISTRY

Supporting Information

for

Benzoimidazolium-derived dimeric and hydride n-dopants for organic electron-transport materials: impact of substitution on structures, electrochemistry, and reactivity

Swagat K. Mohapatra, Khaled Al Kurdi, Samik Jhulki, Georgii Bogdanov, John Bacsa, Maxwell Conte, Tatiana V. Timofeeva, Seth R. Marder and Stephen Barlow

Beilstein J. Org. Chem. **2023,** *19,* 1651–1663. doi:10.3762/bjoc.19.121

Synthetic and other experimental procedures, details of crystal-structure determinations, variable-temperature NMR data, stability data, optical spectra for reactivity studies, and NMR spectra of new compounds

License and Terms: This is a supporting information file under the terms of the Creative Commons Attribution License (https://creativecommons.org/licenses/by/4.0). Please note that the reuse, redistribution and reproduction in particular requires that the author(s) and source are credited and that individual graphics may be subject to special legal provisions.
The license is subject to the *Beilstein Journal of Organic Chemistry* terms and conditions: (https://www.beilstein-journals.org/bjoc/terms)

CONTENTS

1. Synthetic and Other Experimental Procedures S2

2. Details of Crystal-Structure Determinations S7

3. Variable-Temperature NMR Studies of $(N\text{-DMBI})_2$ S10

4. Stability of Dopants S12

5. UV–vis–NIR Spectra of Doping Reactions S15

6. NMR Spectra of New Compounds S16

7. References for Supporting Information S27

1. SYNTHETIC AND OTHER EXPERIMENTAL PROCEDURES

1.1 General. All operations were carried out under an atmosphere of nitrogen using standard Schlenk techniques or in a glove box. Toluene, tetrahydrofuran (THF) and dimethylformamide (DMF) were dried using a solvent purification system from MBraun, benzene and hexane were dried over sodium and distilled, and NEt_3 was stored over KOH and distilled prior to use. Solvents were deoxygenated by three "freeze-pump-thaw" cycle prior to use. All commercially available chemicals were used without further purification unless otherwise noted. Sodium amalgam (1 wt%) was prepared immediately prior to use by addition of small pieces of Na metal to vigorously stirred Hg (electronic grade, 99.99%). 3,6-Dimethoxy-*o*-phenylenediamine (**Ig/h**) [1], (N-DMBI)$_2$ (**1b$_2$**) [2], and 2-cyclohexyl-1,3-dimethyl-2,3-dihydro-1*H*-benzo[*d*]imidazole (**1fH**) [3] were synthesized as described elsewhere. ^{1}H and ^{13}C{^{1}H} NMR spectra were recorded either on Bruker AMX 400 or AVIIIHD 500 MHz spectrometer and were referenced to tetramethylsilane using the residual proton signal of the solvent and the carbon resonances of the deuterated solvent, respectively (see below for spectra). Mass spectra were measured on an Applied Biosystems 4700 Proteomics Analyzer using ESI mode. Elemental analyses were carried out by Atlantic Microlabs using a LECO 932 CHNS elemental analyzer. Electrochemical data were acquired using cyclic voltammetry in 0.1 M ^{n}Bu$_4$NPF$_6$ in dry THF under nitrogen, using a CH Instruments 620D potentiostat, a glassy carbon working electrode, a platinum wire auxiliary electrode, and, as a pseudo-reference electrode, a silver wire anodized in 1 M aqueous potassium chloride solution. A scan rate of 50 mVs^{-1} was used and ferrocene or decamethylferrocene were used as an internal reference. Differential Scanning Calorimetry and Thermal Gravimetric Analysis (see section 3.2 of this Supporting Information) were performed on Metler Toledo instruments under nitrogen gas with heating and cooling rates of 10 °C. Crystal-structure determinations are summarized in Table S1; further details are available in CIF format (see Table S1 for deposition numbers) from the Cambridge Crystallographic Data Center (www.ccdc.cam.ac.uk). UV–vis–NIR kinetic measurements in solution were performed using sealed quartz cuvettes having 1 mm path lengths in a Cary UV–vis–NIR spectrometer. All the stock solutions were prepared in chlorobenzene in a nitrogen-filled glove box. The freshly prepared stock solutions of the respective compounds were mixed in a vial and then transferred to the cuvette for measurements. For the doping experiments involving **1H** derivatives and **VI** (PC$_{61}$BM), the initial concentrations were fixed at 0.4 mM and 2.7 mM, respectively. Doping studies using the dimers were carried out at concentrations for **3$_2$** derivatives and **VI** of 0.2 mM and 2.7 mM, respectively, but were over very rapidly. TIPS-pentacene (**VII**) does not react with **1H** derivatives under the conditions used here. For the studies of reactions between **VII** and **1$_2$** derivatives, the concentrations were 2.6×10^{-5} M and 3.7×10^{-4} M, respectively. Examples of data are shown in Figure S2 (below in section 5).

1.2. Synthesis of Substituted Benzo[*d*]imidazoles (III). A mixture of the appropriate diamine **I** (46.3 mmol), the appropriate aldehyde **II** (46.3 mmol), and sodium metabisulfite (46.3 mmol) were added to a flask with of dry DMF (100 mL). The mixture was heated to 100 °C overnight. The next morning the reaction was cooled down to room temperature and then added to an ice-water

mixture. The white powder was collected via vacuum filtration and dried under high vacuum to afford **III** with quantitative yield.

*2-(4-(Dimethylamino)phenyl)-1H-benzo[d]imidazole, **IIIb***. From **Ib/i** and **Ib/g**. ^{1}H NMR spectra were consistent with a previous report [4].

*2-(4-(Dimethylamino)phenyl)-4,7-dimethoxy-1H-benzo[d]imidazole (**IIIg**)*. From **Ig/h** and **Ib/g**. ^{1}H NMR (400 MHz, methanol-*d*$_4$): δ 7.98 (d, 2H, J = 8 Hz), 6.89 (m, 4H), 4.00 (s, 6H), 3.35 (s, NH, 1H), 3.10 (s, 6H). ^{13}C{^{1}H} NMR (100 MHz, methanol-*d*$_4$): δ 154.86, 151.72, 142.61, 130.48, 125.28, 113.07, 110.31, 106.96, 56.83, 40.25. MS (ESI): *m/z* 298.2 (MH$^+$).

*2-Cyclohexyl-4,7-dimethoxy-1H-benzo[d]imidazole (**IIIh**)*. From **Ig/h** and **Id/h**. ^{1}H NMR (400 MHz, chloroform-*d*): δ 6.52 (s, Ar, 2H), 3.91 (s, 6H), 2.89 (t, cyc, 1H, J = 8 Hz), 2.12 (d, J = 12 Hz, 2H), 1.85 (d, J = 12 Hz, 2H), 1.75-1.58 (m, 3H), 1.44-1.22 (m, 3H). Note, signal corresponding to N*H* signal not observed. ^{13}C{^{1}H} NMR (100 MHz, chloroform-*d*): δ 157.19, 104.34, 101.90, 55.75, 38.51, 31.87, 26.04, 25.98, 25.84. MS (ESI): *m/z* 261.2 (MH$^+$).

*2-(5-(Dimethylamino)-2-thienyl)-1H-benzo[d]imidazole (**IIIi**)*. From **Ib/i** and **Ii**. ^{1}H NMR (400 MHz, acetone-*d*$_6$): δ 7.50 (d, 1H, J = 4 Hz), 7.46 (d, 2H, J = 8 Hz), 7.12 (d, 2H, J = 8 Hz), 5.94 (d, 1H, J = 4 Hz), 3.01 (s, 6H). ^{13}C{^{1}H} NMR (100 MHz, acetone-*d*$_6$): δ 161.6, 184.4, 139.6, 127.2, 121.4, 116.6, 114.0, 102.1, 41.5. HRMS (ESI) calcd for $C_{13}H_{14}N_3S$ (MH$^+$) 244.0903, found 244.0899. Anal. Calcd. for $C_{13}H_{13}N_3S$: C 64.17, H 5.39, N 17.27. Found: C 63.54, H 5.66, N 17.27.

1.3. Synthesis of Imine Derivatives, IV. As noted in the synthesis section of the results and discussion, the attempted synthesis of substituted benzo[*d*]imidazoles in the absence of $Na_2S_2O_5$ sometimes afforded imines rather than the intended compounds. In particular, 4-(((2-amino-3,6-dimethoxyphenyl)imino)methyl)-*N*,*N*-dimethylaniline, **IVg**, was obtained from **Ig/h** and **IIb/g** using reaction conditions adapted from literature reactions involving **Ig/h** and other aldehydes [5, 6]. Specifically **IVg** was obtained as a brownish-yellow solid (4.30 g, 58%) from **Ig/h** (4.00 g, 23.8 mmol) and **IIb/g** (3.54 g, 23.8 mmol) and 1 drop of acetic acid in toluene (65 mL). ^{1}H NMR (500 MHz, chloroform-*d*): δ 8.62 (s, 1H), 7.79 (d, J = 10 Hz, 2H), 6.73 (d, J = 10 Hz, 2H), 6.52 (d, J = 10 Hz, 1H), 6.29 (d, J = 10 Hz, 1H), 3.83 (s, 3H), 3.71 (s, 3H), 3.04 (s, 6H). ^{13}C{^{1}H} NMR (125 MHz, chloroform-*d*): δ 163.5, 152.3, 145.9, 142.1, 132.1, 129.9, 126.7, 111.5, 106.2, 100.2, 56.2, 25.0, 40.2. HRMS (ESI) calcd for $C_{17}H_{20}N_3O_2$ ([M–H]$^+$) 298.1550, found 298.1554; calcd for $C_{15}H_{14}N_3O_2$ ([M–2Me–H]$^+$) 268.1081, found 268.1084 (MH$^+$–2Me).

1.4. Synthesis of DMBI$^+$I$^–$ Salts (1$^+$I$^–$). The corresponding benzimidazole **III** (7.58 mmol), methyl iodide (15.9 mmol) and K_2CO_3 (22.75 mmol) were taken in acetone (75 mL) in a pressure flask. The mixture was stirred at 100 °C for 24 h. The acetone was then removed using rotary evaporation. The resulting solids were in some cases used for metathesis to the corresponding hexafluorophosphates without further purification, but in most cases (see below) were also purified

by extraction into dichloromethane, evaporation, and recrystallization from dichloromethane / diethyl ether.

*2-(4-(Dimethylamino)phenyl)-1,3-dimethylbenzo[d]imidazolium iodide (**1b**$^+$**I**$^-$).* Off-white solid (73%). ^{1}H NMR spectra were consistent with a previous report [4].

*2-(4-(Dimethylamino)phenyl)-4,7-dimethoxy-1,3-dimethylbenzo[d]imidazolium iodide (**1g**$^+$I$^-$).* Light yellow/orange solid (74 %). ^{1}H NMR (500 MHz, acetone-d_6): δ 7.75 (d, J = 9 Hz, 2H), 7.15 (s, 2H), 7.05 (d, J = 9 Hz, 2H), 4.13 (s, 6H), 4.05 (s, 6H), 3.16 (s, 6H). ^{13}C{^{1}H} NMR (125 MHz, acetone-d_6): δ 153.8, 152.8, 143.0, 132.8, 124.1, 112.6, 108.6, 106.6, 57.2, 40.0, 36.4. HRMS (ESI): calcd for $C_{19}H_{24}N_3O_2$ ([M–I]$^+$) 326.1863, found, 326.1857;calcd for $C_{17}H_{18}N_3O_2$ ([M–2Me–I]$^+$) 296.1394, found 296.1386. Anal. Calcd. for $C_{19}H_{24}N_3O_2I$: C 50.34, H 5.34, N 9.27; found: C 49.80, H 5.49, N 9.30.

*2-Cyclohexyl-4,7-dimethoxy-1,3-dimethylbenzo[d]imidazolium iodide (**1h**$^+$I$^-$).* White solid (78%). ^{1}H NMR (400 MHz, chloroform-d): δ 6.84 (s, 2H), 4.43 (s, 6H), 3.96 (s, 6H), 3.64−3.56 (m, 1H), 2.28−2.25 (m, 2H), 2.01−1.89 (m, 5H), 1.72−1.68 (m, 2H), 1.45−1.34 (m, 1H). ^{13}C{^{1}H} NMR (100 MHz, chloroform-d): δ 154.3, 142.1, 123.1, 107.631, 56.6, 36.6, 36.1, 28.8, 26.0, 25.3. HRMS (ESI): calcd for $C_{17}H_{25}N_2O_2$ ([M–I]$^+$) 289.1911, found 289.1909; calcd for $C_{15}H_{19}N_2O_2$ ([M–2Me–I]$^+$) 259.1441, found 259.1438. Anal. Calcd. for $C_{17}H_{25}N_2O_2I$: C 49.05, H 6.05, N 6.73; found: C 49.17, H 5.97, N 6. 60.

1.5. Synthesis of DMBI$^+$PF$_6^-$ Salts (1$^+$PF$_6^-$). The iodide salt, **1**$^+$I$^-$ (1.25 mmol), was dissolved in a minimum of methanol. To it an excess of NH_4PF_6 (6.1 mmol) was added; the mixture was sonicated for 5 min and then copious amounts of deionized water were added. The product was collected via vacuum filtration, washed with water, and dried under vacuum.

*2-(4-(Dimethylamino)phenyl)-1,3-dimethylbenzo[d]imidazolium hexafluorophosphate (**1b**$^+$PF$_6^-$).* White solid (93%). ^{1}H and ^{13}C{^{1}H} NMR spectra were consistent with our previous report [2].

*2-(4-(Dimethylamino)phenyl)-4,7-dimethoxy-1,3-dimethylbenzo[d]imidazolium hexafluorophosphate (**1g**$^+$PF$_6^-$).* Light yellow solid (89%). ^{1}H NMR (500 MHz, acetone-d_6): δ 7.66 (d, J = 9 Hz, Ar, 2H), 7.11 (s, 2H), 7.03 (d, J = 9 Hz, 2H), 4.10 (s, 6H), 4.02 (s, 6H), 3.14 (s, 6H). ^{13}C{^{1}H} NMR (125 MHz, acetone-d_6): δ 154.0, 152.9, 143.1, 132.7, 124.2, 112.7, 108.6, 106.6, 57.2, 40.1, 36.5. Anal. Calcd. for $C_{19}H_{24}N_3O_2PF_6$: C 48.41, H 5.13, N 8.91. Found C 48.14, H 5.09, N 8.75.

*2-Cyclohexyl-4,7-dimethoxy-1,3-dimethylbenzo[d]imidazolium hexafluorophosphate (**1h**$^+$PF$_6^-$).* White solid (95%). ^{1}H NMR (500 MHz, acetone-d_6): δ 7.07 (s, 2H), 4.41 (s, 6H), 4.00 (s, 6H), 3.67 (m, 1H), 1.44−1.38 (m, 4H), 1.96−1.93 (m, 2H), 1.81−1.79 (m, Cyc, 1H), 1.60−1.58 (m, 3H).

$^{13}C\{^1H\}$ NMR (125 MHz, acetone-d_6): δ 155.4, 143.0, 124.0, 108.6, 57.1, 36.5, 36.0, 28.3, 26.7, 25.9. Anal. Calcd. for $C_{17}H_{25}N_2O_2PF_6$: C 47.01, H 5.80, N 6.45. Found C 47.29, H 5.60, N 6.63.

*2-(5-(Dimethylamino)-2-thienyl)-4,7-dimethoxy-1,3-dimethylbenzo[d]imidazolium hexafluorophosphate (**1i**$^+$$PF_6^-$).* White solid (95%) from **IIIi** without purification or characterization of **1i**$^+$I$^-$ intermediate. ^1H NMR (400 MHz, acetone-d_6): δ 7.98 (dd, J = 2.4, 4 Hz), 7.82 (d, J = 4 Hz, 1H), 7.72 (dd, J = 2.4, 4 Hz, 2H), 6.34 (d, ,J = 4 Hz, 1H), 4.23 (s, 6H), 3.22 (s, 6H). $^{13}C\{^1H\}$ NMR (100 MHz, acetone-d_6): δ 167.18, 139.13, 132.45, 126.33, 112.40, 103.22, 68.31, 53.79, 41.67, 33.07. HRMS (ESI): calcd for $C_{15}H_{18}SN_3$ ([M–PF_6]$^+$) 272.1216, found 272.1215. Anal. Calcd. for $C_{15}H_{18}F_6N_3PS$: C 43.17, H 4.35, N 10.07; found C 43.42, H 4.54, N 10.17.

1.6. Synthesis of DMBI-H Derivatives (1H). To a solution of **1**$^+$I$^-$ or **1**$^+$$PF_6^-$ (2.5 mmol) in MeOH (20 mL), solid $NaBH_4$ (7.62 mmol) was added in small portions over ca. 10 min. The mixture was stirred at room temperature for 1 h; then the solution was concentrated by rotary evaporation, and then water was added to precipitate the product. The solid was collected by filtration, washed with little water, and dried under high vacuum. The materials were further purified if necessary by dissolution in benzene, filtration, and evaporation.

*2-(4-(Dimethylamino)phenyl)-1,3-dimethyl-2,3-dihydro-1H-benzo[d]imidazole (N-DMBI-H, **1bH**).* White solid (81% from **1b**$^+$I$^-$), ^1H and $^{13}C\{^1H\}$ NMR spectra for which were consistent with previous reports [7-9].

*2-(4-(Dimethylamino)phenyl)-4,7-dimethoxy-1,3-dimethyl-2,3-dihydro-1H-benzo[d]imidazole (**1gH**).* Light gray solid (67% from **1g**$^+$I$^-$). ^1H NMR (400 MHz, benzene-d_6): δ 7.58 (d, J = 8.4 Hz, 2H), 6.60 (d, J = 8.8 Hz, 2H), 6.32 (s, 2H), 4.66 (s, 1H), 3.42 (s, 6H), 2.95 (s, 6H), 2.48 (s, 6H). $^{13}C\{^1H\}$ NMR (100 MHz, benzene-d_6): δ 151.3, 141.7, 131.5, 130.0, 127.2, 112.2, 105.7, 96.4, 56.0, 39.8, 36.2. MS(ESI): m/z 296.1 ([M–2Me–H]$^+$). Anal. Calcd. for $C_{19}H_{25}N_3O_2$: C 69.70, H 7.70, N 12.8; found C 69.63, H 7.56, N 12.73.

*2-Cyclohexyl-4,7-dimethoxy-1,3-dimethyl-2,3-dihydro-1H-benzo[d]imidazole (**1hH**).* White solid (63% from **1h**$^+$I$^-$). ^1H NMR (500 MHz, C_6D_6): δ 6.28 (s, 1H), 3.75 (d, J = 5Hz, 1H), 3.45 (s, 6H), 2.91 (s, 6H), 1.85−1.82 (m, 2H), 1.70−1.68 (m, 2H), 1.60 (m, 1H), 1.45−144 (m, 1H), 1.18−1.12 (m, 5H). $^{13}C\{^1H\}$ NMR (125 MHz, C_6D_6): δ 142.9, 133.6, 105.9, 97.5, 55.8, 44.9, 42.1, 27.9, 27.1, 26.7. HRMS (ESI): calcd for $C_{15}H_{19}N_2O_2$ ([M–2Me–H]$^+$) 259.1441, found 259.1437. Anal. Calcd. for $C_{17}H_{26}N_2O_2$: C 70.31, H 9.02, N 9.65. Found C 69.73, H 8.80, N 9.60.

*2-(5-(Dimethylamino)-2-thienyl)-4,7-dimethoxy-1,3-dimethyl-2,3-dihydro-1H-benzo[d]imidazole (**1iH**).* White solid (97% from **1i**$^+$$PF_6^-$). ^1H NMR (400 MHz, chloroform-d): δ 6.90 (d, J = 4 Hz, 1H), 6.73-6.68 (m, 2H), 6.46-6.41 (m, 2H), 5.73 (d, J = 4 Hz, 1H), 4.92, (s, 1H), 2.93 (s, 6H), 2.64 (s, 6H). $^{13}C\{^1H\}$ NMR (100 MHz, chloroform-d): δ 160.70, 141.65, 128.22, 127.10, 119.35,

105.98, 100.13, 90.26, 42.64, 33.12. HRMS (ESI) calcd. for $C_{15}H_{20}N_2S$ (MH^+) 274.1372, found 274.1372. Anal. Calcd. for $C_{15}H_{19}N_3S$: C 65.90, H 7.01, N 15.37 Found C 65.64, H 6.96, N 15.14.

1.7. Synthesis of (Y-DMBI)$_2$ Derivatives (1$_2$). 1 wt % Na:Hg was made by slow addition of freshly cut and washed sodium pieces (400 mg, 17 mmol) to vigorously stirred mercury (electronic grade, 99.99%, 40 g) under nitrogen. A slurry of **1**$^+PF_6^-$ (2.3 mmol) in dry THF was stirred over this amalgam for 2 h at room temperature under nitrogen, during which time all the solids dissolved in the solution and the color was slightly changed. The mixture was then decanted from the amalgam via cannula and evaporated under reduced pressure. The crude product was again extracted in toluene, decanted carefully to minimize the insoluble solid, and then filtered using syringe filters inside a glove box (alternatively the solution can be filtered through Celite). The solution was evaporated under reduced pressure, and further washed with hexane to obtain a pure solid. If required, further purification was carried out by recrystallization cold hexane or by simply quickly rinsing with the same solvent.

*2,2'-Di(4-(dimethylamino)phenyl)-1,1',3,3'-tetramethyl-2,2',3,3'-tetrahydro-1H,1'H-2,2'-bibenzo[d]imidazole ((N-DMBI)$_2$, **1b$_2$**).* The synthesis and characterization of **1b$_2$** according to a method similar to the general procedure described above has been previously reported [2]. Larger quantities of the dimer can be obtained while minimizing the use of toxic and costly Hg by carrying out the reduction in batches: the reaction is carried out as described above and after 90–120 min the supernatant is decanted via canula, and the resulting amalgam washed with a small quantity of THF before addition of fresh **1b**$^+PF_6^-$ and THF. This process can be carried out several times. The Hg can be used for yet more batches by addition of fresh sodium. Additional variable-temperature NMR data is shown in section S3.1.

*2,2'-Di(4-(dimethylamino)phenyl)-4,4',7,7'-tetramethoxy-1,1',3,3'-tetramethyl-2,2',3,3'-tetrahydro-1H,1'H-2,2'-bibenzo[d]imidazole (**1g$_2$**).* Light gray solid (74%). ^{1}H NMR (400 MHz, benzene-d_6): δ 7.81 (apparent d, J_{app} = 6.0 Hz, 2H), 6.45-6.32 (br, m, 8H), 6.20 (d, J = 8.8 Hz, 2H), 3.48 (s, 6H), 3.36 (s, 6H), 3.16 (s, 6H), 3.14 (s, 6H), 2.47 (s, 12H). ^{13}C{^{1}H} NMR (100 MHz, benzene-d_6): δ 149.5, 141.0, 138.9, 134.7, 132.7, 132.2, 125.4, 110.4, 106.4, 103.1, 98.5, 59.6, 56.4, 39.5, 37.9, 36.4. HRMS (ESI): calcd for $C_{17}H_{18}N_3O_2$ ($[M/2–2Me]^+$) 296.1394, found 296.1391. Anal. Calcd. for $C_{38}H_{48}N_6O_4$: C 69.91, H 7.41, N 12.87. Found C 69.79, H 7.54, N 12.70.

*2,2'-Dicyclohexyl-4,4',7,7'-tetramethoxy-1,1',3,3'-tetramethyl-2,2',3,3'-tetrahydro-1H,1'H-2,2'-bibenzo[d]imidazole (**1h$_2$**).* White solid (79%). ^{1}H NMR (500 MHz, benzene-d_6): δ 6.25 (s, 4H), 3.47 (s, 12H), 3.44 (s, br, 12H), 2.16 (m, 2H), 1.93 (br, 4H), 1.61−1.58 (m, 6H), 1.41−1.38 (m, 4H), 1.17−1.05 (br, 6H). ^{13}C{^{1}H} NMR (125 MHz, benzene-d_6): δ 139.0, 131.6, 105.9, 98.1, 57.5, 47.9, 35.5, 29.9, 28.4, 27.3. HRMS (ESI): calcd for $C_{15}H_{19}N_2O_2$ ($[M/2–2Me]^+$) 259.1441, found 259.1436. Anal. Calcd. For $C_{34}H_{50}N_4O_4$: C 70.56, H 8.71, N 9.68. Found C 70.94, H 8.70, N 9.55.

2. DETAILS OF CRYSTAL-STRUCTURE DETERMINATIONS

Experimental details of the crystal-structure determinations are presented in Table S1. Some geometric characteristics of the molecules and cations are compared in Table S2, along with those of some other related **1**$_2$, **1H**, and **1**$^+$X$^-$ derivatives. Figure S1 defines the geometric parameters summarized in Table S2

Table S1. Crystal Data and Structural Refinement Parameters.

	**1b**$_2$	**1h**$_2$	**1bH**	**1gH**	**1hH**	**1iH**	**1g**$^+$**I**$^-$	**1h**$^+$**PF**$_6^-$	**1i**$^+$PF$_6^-$
CCDC #	2192097	2192588	2192118	2269184	2269185	2269187	2269183	2269182	2269186
Diffractometer	Bruker D8 Venture	Bruker D8 Venture	XtaLAB Synergy	Bruker Apex-II CCD	Bruker Apex-II CCD	Bruker Apex-II CCD	Bruker Apex-II CCD	Bruker Apex-II CCD	Bruker Apex-II CCD
Emp. formula	$C_{34}H_{40}N_6$	$C_{34}H_{50}N_4O_4$	$C_{17}H_{21}N_3$	$C_{19}H_{25}N_3O_2$	$C_{17}H_{26}N_2O_2$	$C_{15}H_{19}N_3S$	$C_{19}H_{24}IN_3O_2$	$C_{17}H_{25}F_6N_2O_2P$	$C_{15}H_{18}F_6N_3PS$
FW	532.737	578.801	267.376	327.42	290.40	273.39	453.31	434.36	417.35
T / K	100	100	112	150	150	150	150	150	150
Crystal system	triclinic	monoclinic	Monoclinic	monoclinic	monoclinic	orthorhombic	monoclinic	monoclinic	triclinic
Space group	$P\bar{1}$	$P2_1/n$	$P2_1/n$	$P2_1/c$	$P2_1/c$	$P2_12_12_1$	$P2_1/c$	$P2_1/n$	$P\bar{1}$
a / Å	12.093(2)	9.3458(13)	14.2283(11)	14.88(2)	15.545(3)	9.0614 (2)	13.017(4)	10.1709(13)	7.4425(15)
b / Å	12.874(3)	20.593(3)	8.1681(4)	6.897(11)	6.5898(12)	12.3962(3)	7.521(2)	19.090(2)	8.5626(5)
c / Å	14.791(3)	15.755(2)	14.4641(11)	17.71(3)	15.483(3)	12.5311(3)	21.557(7)	10.2168(13)	14.4366(9)
α / °	71.064(7)	90	90	90	90	90	90	90	104.726(2)
β / °	80.545(7)	98.966(5)	117.283(10)	101.494(14)	91.852(2)	90	98.188(4)	98.208(2)	94.310(3)
γ / °	85.837(7)	90	90	90	90	90	90	90	96.293(3)
Volume / Å^3	2148.1(8)	2995.0(7)	1494.0(2)	1781(5)	1585.3(5)	1408.00(6)	2088.8(11)	1963.4(4)	879.33(10)
Z	3	4	4	4	4	4	4	4	2
ρ_{calc} / g cm^{-3}	1.235	1.284	1.189	1.221	1.217	1.290	1.441	1.469	1.576
μ / mm^{-1}	0.075	0.085	0.072	0.080	0.080	0.218	1.549	0.211	0.340

F(000)	858	1256	576	704	632	584	912	904	428
Crystal size / mm^3	0.28 × 0.24 × 0.13	0.41 × 0.29 × 0.19	0.33 × 0.16 × 0.13	0.24 × 0.18 × 0.15	0.3 × 0.25 × 0.1	0.32 × 0.2 × 0.15	0.34 × 0.25 × 0.11	0.3 × 0.15 × 0.1	0.23 × 0.16 × 0.12
Crystal color / shape	colorless prism	colorless prism	colorless needle	green block	white block	yellow block	colorless prism	colorless prism	gray block
Radiation (λ / Å)	MoK_α (0.71073)	MoK_α (0.71073)	MoK_α (0.71073)	MoK_α (0.71073)	MoK_α (0.71073)	MoK_α (0.71073)	MoK_α (0.71073)	MoK_α (0.71073)	MoK_α (0.71073)
2θ range / °	4.66-80.58	4.74-66.32	3.32-67.58	2.31-52.55	4.60-33.11	1.58-26.61	4.67- 33.31	2.13-31.87	1.47-56.07
Index ranges	$-21 \le h \le 21$<br>$-23 \le k \le 23$<br>$-26 \le l \le 26$	$-14 \le h \le 14$<br>$-31 \le k \le 31$<br>$-22 \le l \le 24$	$-21 \le h \le 22$<br>$-12 \le k \le 12$<br>$-21 \le l \le 22$	$-20 \le h \le 18$<br>$-27 \le k \le 26$<br>$-27 \le l \le 27$	$-22 \le h \le 22$<br>$-10 \le k \le 10$<br>$-26 \le l \le 26$	$-15 \le h \le 16$<br>$-9 \le k \le 9$<br>$-26 \le l \le 27$	$-23 \le h \le 22$<br>$-10 \le k \le 10$<br>$-23 \le l \le 23$	$-14 \le h \le 14$<br>$-28 \le k \le 26$<br>$-14 \le l \le 14$	$-16 \le h \le 16$<br>$-19 \le k \le 19$<br>$-33 \le l \le 33$
Total refs	73245	55099	46855	41277	33849	19929	38723	22895	197680
Ind. refs (R_{int})	26873(0.0541)	1133(0.0773)	5546(0.0409)	16061(0.0739)	6624(0.1456)	4360(0.0394)	6042(0.0488)	6230(0.0502)	22591(0.0638)
Completness to θ=25.2417°	99.9%	99.6%	99.9%	95.9%	99.3%	99.9%	99.3%	100%	100%
Data / restr'ts / params	26873/705/1082	11337/711/588	5546/0/370	16061/0/176	6624/0/224	6230/0/313	6042/0/194	22591/0/239	4360/0/232
Goodness-of-fit on F^2	1.0397	1.0667	1.0943	1.003	0.992	1.163	1.034	1.051	1.140
Final R indices [$I \ge 2\sigma(I)$]	R_1 = 0.0463<br>wR_2 = 0.0989	R_1 = 0.0509<br>wR_2 = 0.1073	R_1 = 0.0170<br>wR_2 = 0.0287	R_1 = 0.0623<br>wR_2 = 0.1415	R_1 = 0.0625<br>wR_2 = 0.1442	R_1 = 0.0421<br>wR_2 = 0.937	R_1 = 0.0460<br>wR_2 = 0.1205	R_1 = 0.0609<br>wR_2 = 0.1753	R_1 = 0.0544<br>wR_2 = 0.1289
Final R indices [all data]	R_1 = 0.0725<br>wR_2 = 0.1168	R_1 = 0.0724<br>wR_2 = 0.1216	R_1 = 0.0227<br>wR_2 = 0.0295	R_1 = 0.0984<br>wR_2 = 0.1625	R_1 = 0.1367<br>wR_2 = 0.1800	R_1 = 0.0508<br>wR_2 = 0.0973	R_1 = 0.0648<br>wR_2 = 0.1366	R_1 = 0.0866<br>wR_2 = 0.1981	R_1 = 0.0948<br>wR_2 = 0.1631
Largest peak; hole / eÅ^{-3}	0.39; −0.49	0.71; −0.67	0.13; −0.13	1.756; −1.204	0.258; −0.283	1.295; −1.005	0.451; −0.217	0.298; −0.313	0.589; −0.952

Table S2. Selected Geometric Parameters[a] from Crystal structures of $(DMBI)_2$, DMBI-H and $DMBI^+$ Compounds.

Structure	Symmetry[b]	C^2–$C^{2'}$ / Å	av. $\Sigma\theta$ / °	ϕ / °	χ / °	ψ / °	ω / °
$\mathbf{1b_2}$[c]	C_i (C_{2h})	1.5899(13)	344.2	16.1	89.4	36.6, 36.9	91.7, 91.8
	C_1	1.6194(8)	352.3	1.0, 17.1	75.2, 71.7	54.9, 30.3, 50.5, 6.1	98.1, 76.6, 76.7, 134.7
$\mathbf{1c_2}$[cd]	C_i	1.595(5)	350.1	6.4	86.4	24.6, 50.0	80.4, 101.7
	C_i	1.601(5)	350.2	5.7	87.4	25.7, 45.6	84.7, 100.9
$\mathbf{1e_2}$[d]	C_2	1.640(4)	355.5	6.9	–	44.0, 46.7	78.5, 83.9
$\mathbf{1h_2}$	C_1 (C_2)	1.6299(13)	352.8	9.7, 6.3	–	81.6, 86.9, 84.9, 90.9	41.3, 40.2, 36.0, 38.1
**1bH**	C_1	–	341.3	31.8	83.2	67.2, 74.1	47.7, 53.6
**1cH**[ce]	C_1	–	340.0	24.2	89.4	73.2, 89.0	35.6, 46.5
	C_1	–	343.1	30.8	71.3	67.4, 76.1	49.2, 53.5
**1dH**[ce]	C_1	–	340.7	25.4	85.7	73.0, 85.1	39.6, 46.9
	C_1	–	342.6	29.2	75.3	69.4, 77.7	47.2, 51.2
**1gH**	C_1	–	337.2	30.2	79.7	75.2,76.2	44.2, 47.0
**1hH**	C_1	–	338.0	19.0	–	83.4, 90.4	31.8, 36.4
**1iH**	C_1	–	339.1	32.4	80.6	73.0, 72.8	49.6, 48.9
$\mathbf{1b}^+PF_6^-$[f]	C_1 (C_2)	–	359.7	0.3	52.5	5.6, 5.9	–
$\mathbf{1c}^+BAr'_4{}^-$[ceg]	C_1	–	359.7	2.1	33.1	3.4, 12.8	–
	C_1	–	359.6	0.4	40.0	3.0, 6.1	–
$\mathbf{1d}^+BAr'_4{}^-$[eg]	C_1	–	359.8	0.3	37.9	1.9, 5.6	–
$\mathbf{1g}^+I^-$	C_1 (C_2)	–	359.7	0.7	41.5	0.4, 11.1	–
$\mathbf{1h}^+PF_6^-$	C_1	–	360.0	0.6	–	0.7, 0.8	–
$\mathbf{1i}^+PF_6^-$	C_1	–	359.7	0.7	31.9	0.6, 22.3	–

[a] See Fig.S1 for definitions of parameters. [b] Point group corresponding to crystallographic symmetry of the molecule or cation (approximate symmetry of molecule or cation in parentheses where this is higher than the crystallographic symmetry). [c] These structures contain two crystallographically inequivalent molecules. [d] From ref. [10]. [e] From ref. [11]. [f] From ref. [2]. [g] Ar' = 3,5-$(CF_3)C_6H_3$.

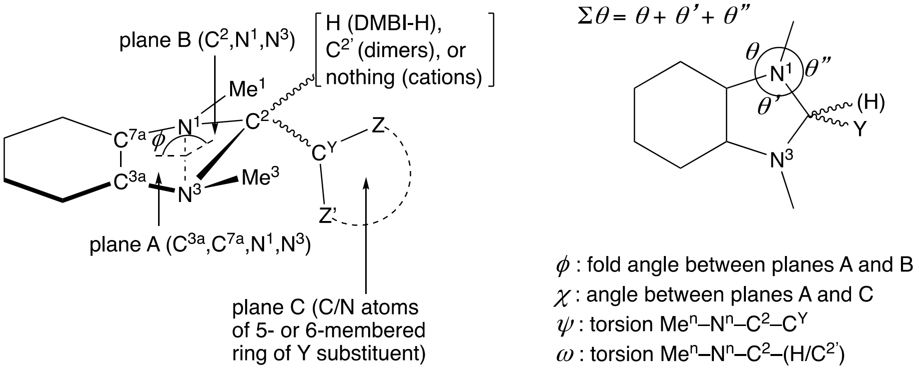

Fig. S1. Definition of structural parameters used to compare **1**$_2$, **1H**, and **1**$^+$ structures in Table S2.

3. Variable-Temperature NMR Studies of (N-DMBI)$_2$

We have previously noted that the 500 MHz ^{1}H NMR spectrum of **1b**$_2$ at room temperature is characterized by broad features [2]. Moreover, the spectrum is more complex than expected if the dimer has the highest possible (C_{2h}) symmetry; for example, there are five distinct signals in the range 2.36-2.66 ppm, attributable to methyl groups. Fig. S2 shows variable temperature (283–343 K) ^{1}H NMR data for **1b**$_2$. As the temperature is raised some of the resonances broaden and coalesce, with some becoming sharper at still higher temperatures. At the highest temperature examined the number of resonances matches that for the high-symmetry conformer (4 peaks in the aromatic region and 2 in the aliphatic region), although the peaks are still somewhat broad, especially the most strongly downfield of the aromatic resonances and the aliphatic signal assigned to an imidazole NMe resonance. These data strongly suggest that there is restricted rotation in this compound at room temperature and that, even at 343 K, the rates of rotation are still insufficient to completely average some of these resonances.

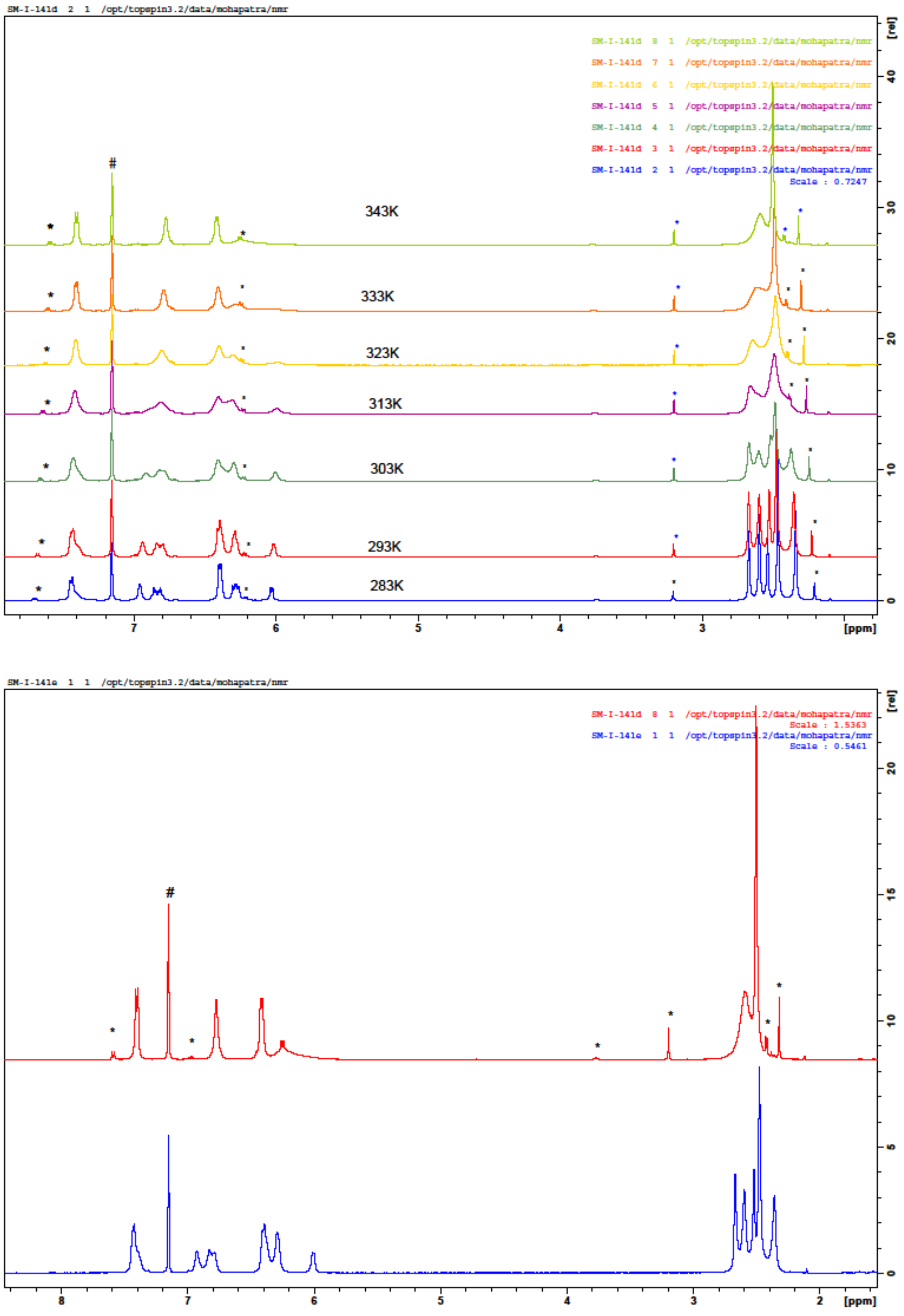

Fig. S2. Top: ^{1}H NMR spectra of **1b**$_2$ in C_6D_6 at various temperatures. Bottom: Vertically expanded spectra for 298 and 343 K. Note that this sample contains traces of the amide side / decomposition product **Vb**, signals attributed to which are marked *. The signal marked # corresponds to the residual protons in the solvent.

3. Stability of Dopants

3.1. NMR Studies of Dopant Stability. When an NMR tube containing a C_6D_6 solution of **1b**$_2$ was exposed to air for few minutes, then capped and left for 24 h at room temperature, the signals attributable to the dimer were found to disappear, those of the amide **Vb** were found to increase, and a small quantity of white solid, presumably **1b**$^+$OH$^-$, was observed (Fig. S3). On the other hand, the solid dopant is stable in inert atmosphere for at least 4 months. Amide **Vb** was also observed on exposure of a C_6D_6 solution of **1bH** to air (Fig. S4), consistent with other recent work [9]. We did not compare the stability of all the dopants examined; however, **1hH** was found to be considerably more stable in air-exposed solution than **1bH** (Fig. S5), qualitatively consistent with its lower reactivity with $PC_{61}BM$.

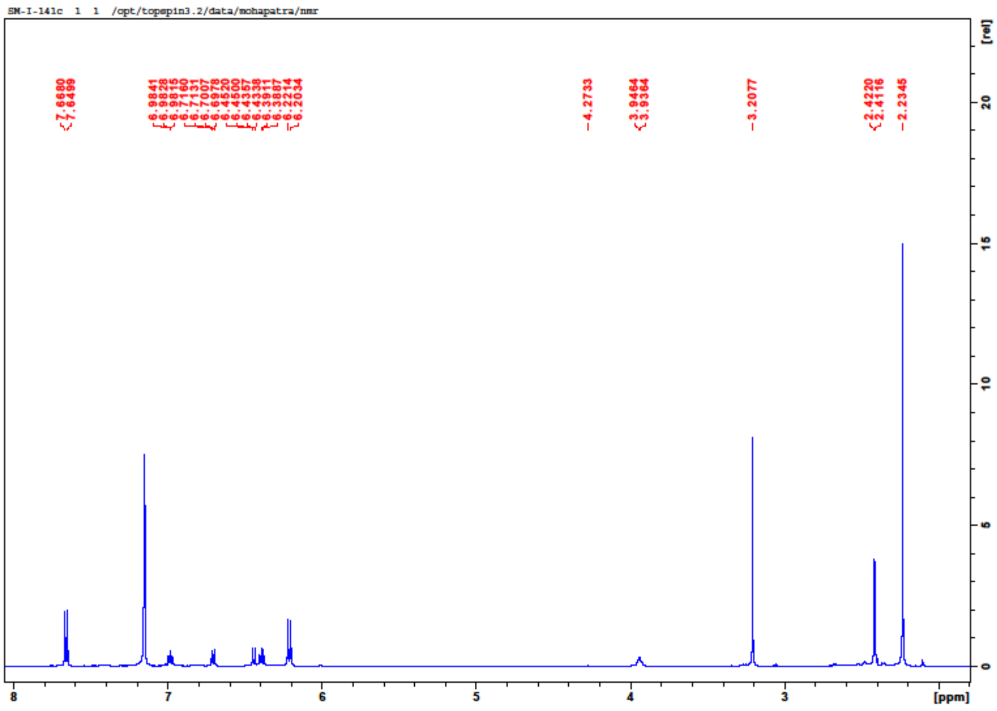

Fig. S3. ^{1}H NMR spectrum of pure **Vb** in C_6D_6 obtained by exposing a sample of **1b**$_2$ to air and storing for 24 h.

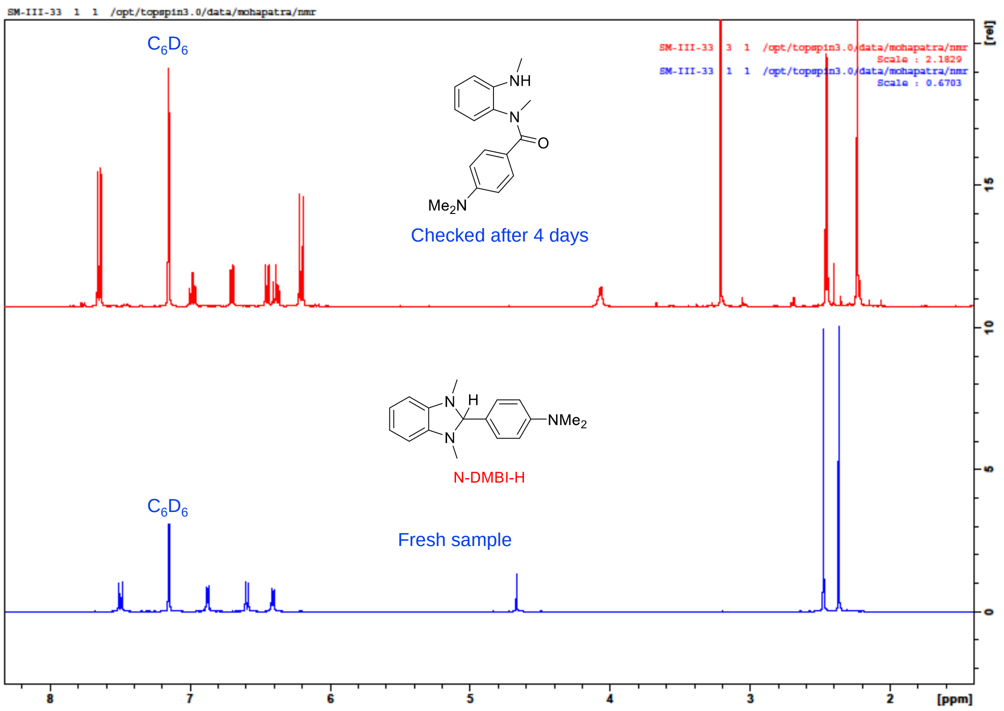

Fig. S4. Bottom and top panels show before and after ^{1}H NMR spectra of a **1bH** sample in C_6D_6 exposed to air and stored for 4 days, resulting in formation of **Vb**.

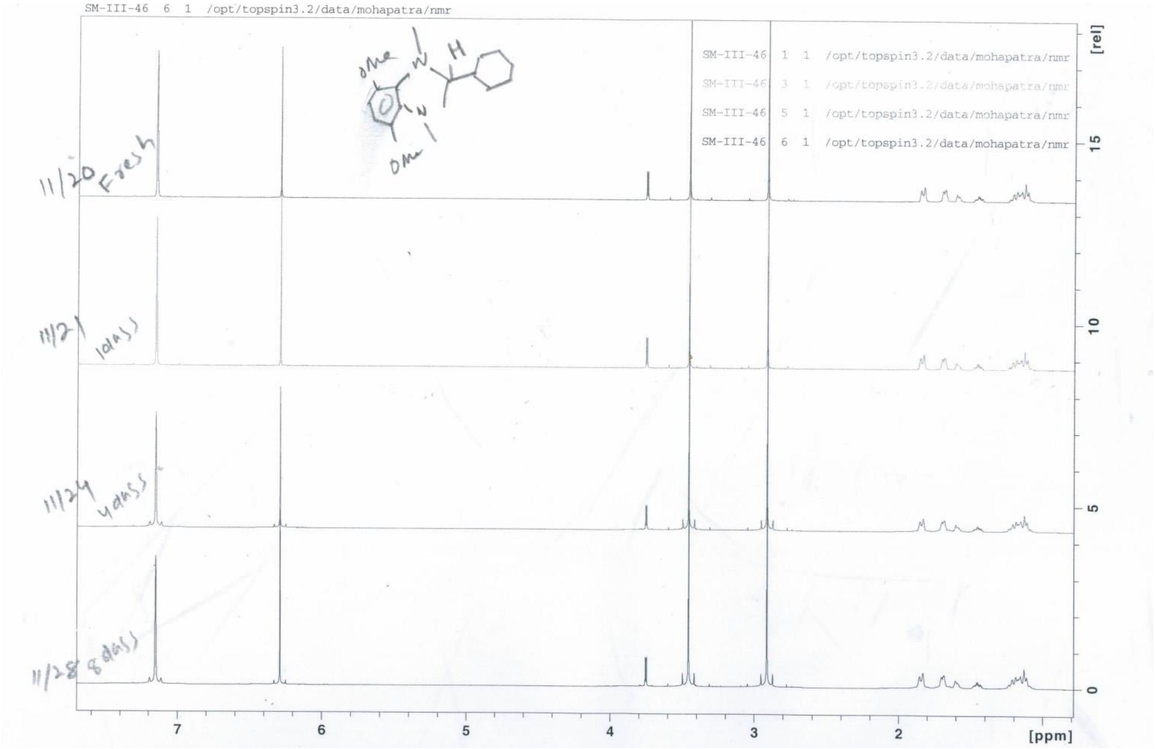

Fig. S5. ^{1}H NMR spectra of a **1hH** sample in C_6D_6; from top to bottom before and 1, 4, and 8 days after exposure to air, showing this dopant to be considerably more air stable than **1bH** (Fig. S4).

3.2. Thermal Characterization.

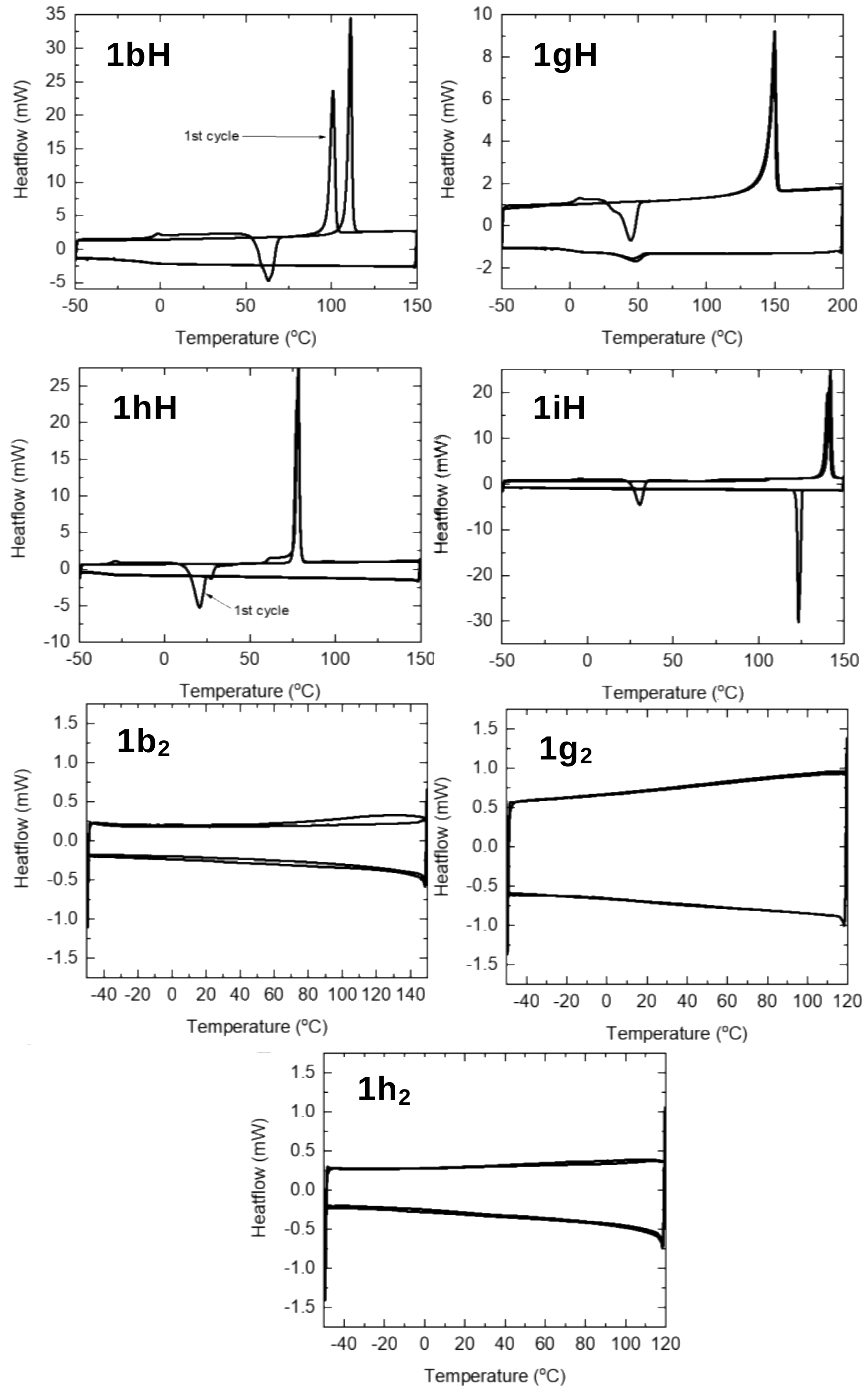

Fig. S6. Differential scanning calorimetry data (10 °C min^{-1}) for **1H** and **1**$_2$ derivatives.

Table S3. Thermal Data for 1H and 1_2 Derivatives.

	TGA[a]	DSC[b]		
Compound	T_d / °C	T_g / °C	T_{cryst} / °C	T_{melt} / °C
**1bH**	191	0	65	100
**1gH**	240	0	50	150
**1hH**	173	–30	23	75
**1iH**	221	0	25	140
**$1b_2$**	186	*c*	*c*	*c*
**$1g_2$**	170	*c*	*c*	*c*
**$1h_2$**	219	*c*	*c*	*c*

[a] Defined as temperature at 5% weight loss is observed on heating at 10 °C min^{-1}. [b] Glass transition temperature (T_g), exotherms assigned to cold crystallization (T_{cryst}), and melting points (T_{melt}) observed in first heating scan of DSC at a heating rate of 10 °C min^{-1}. [c] No features observed.

5. UV–Vis–NIR Spectra of Doping Reactions

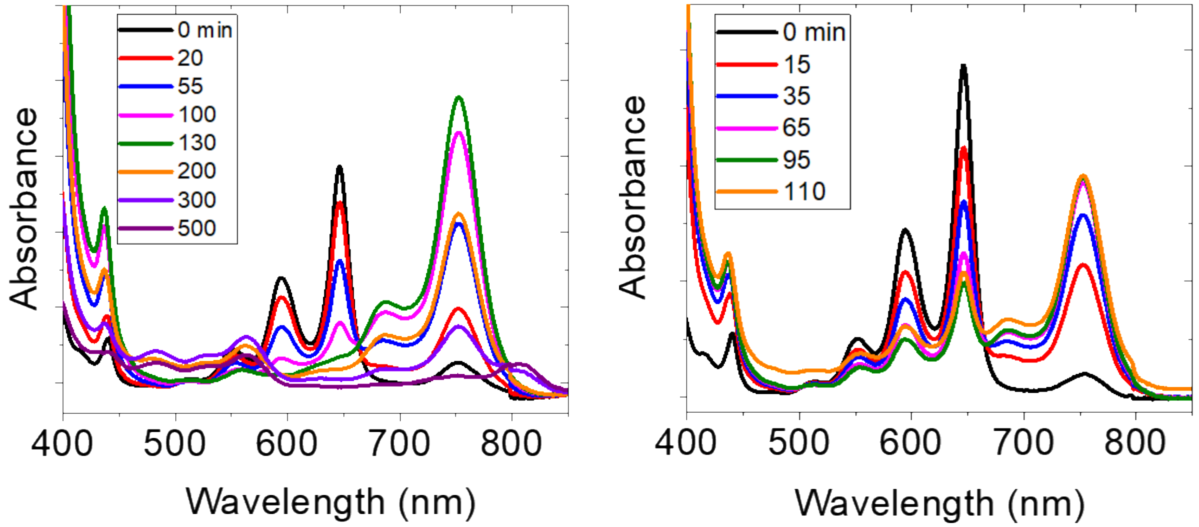

Fig. S7. Examples of vis.-NIR spectra obtained during the reaction of **VII** with excess **$1g_2$** (left) and **$1h_2$** (right).

6. NMR Spectra of New Compounds

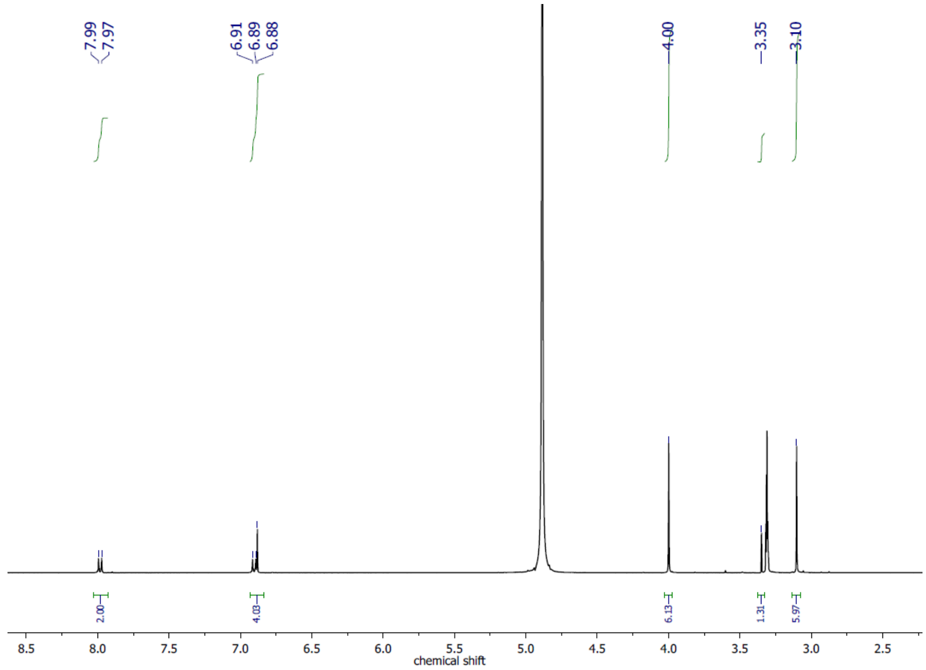

Fig. S8. ^{1}H NMR Spectrum of **IIIg** in CD_3OD.

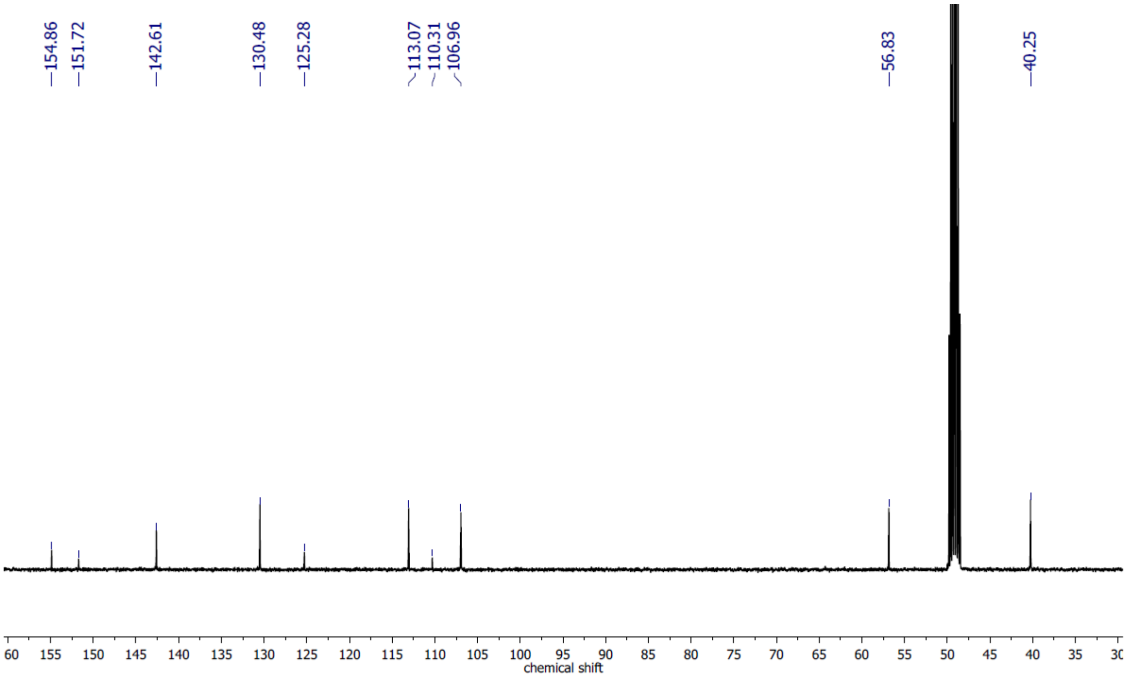

Fig. S9. $^{13}C\{^{1}H\}$ NMR Spectrum of **IIIg** in CD_3OD.

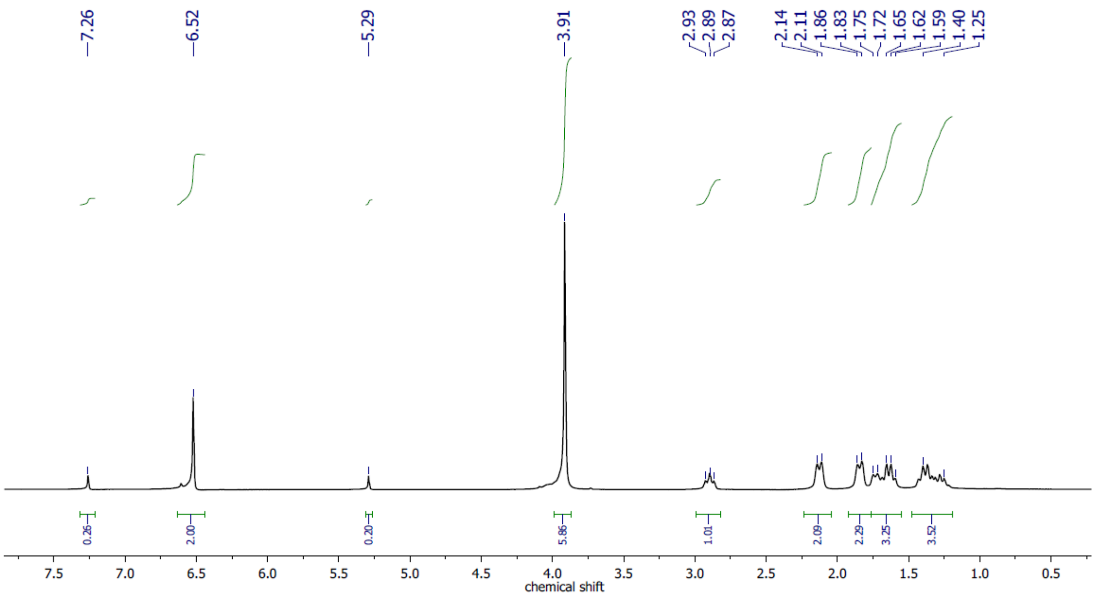

Fig. S10. ^{1}H NMR Spectrum of **IIIh** in $CDCl_3$.

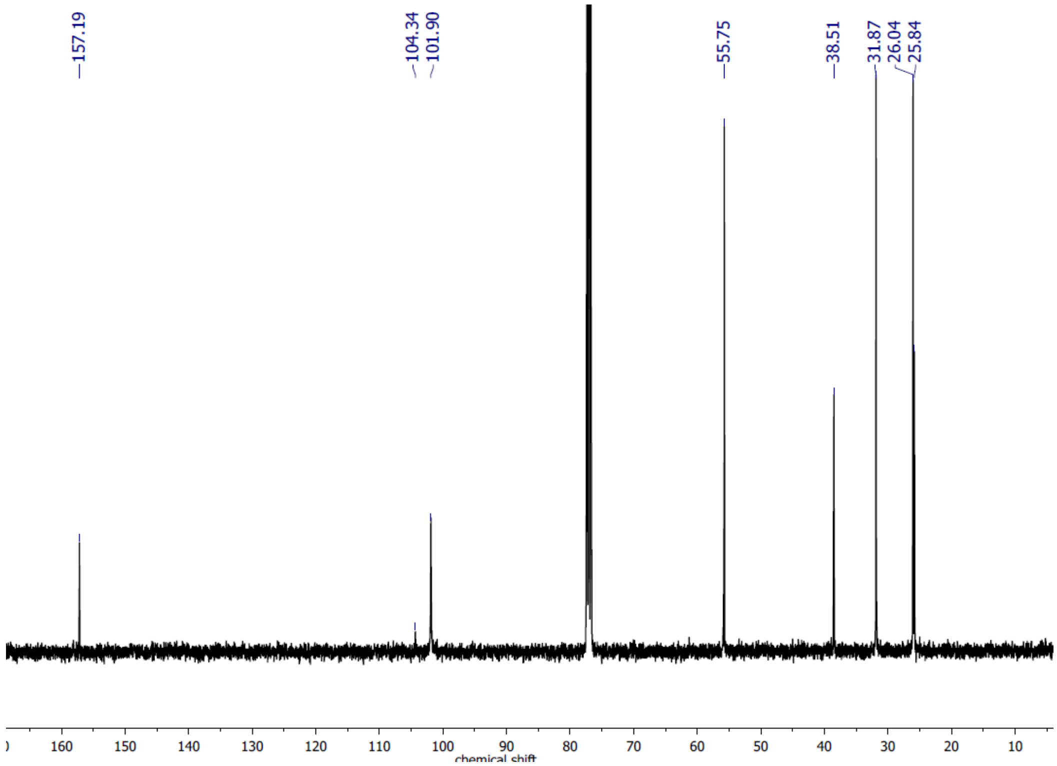

Fig. S11. $^{13}C\{^{1}H\}$ NMR Spectrum of **IIIh** in $CDCl_3$.

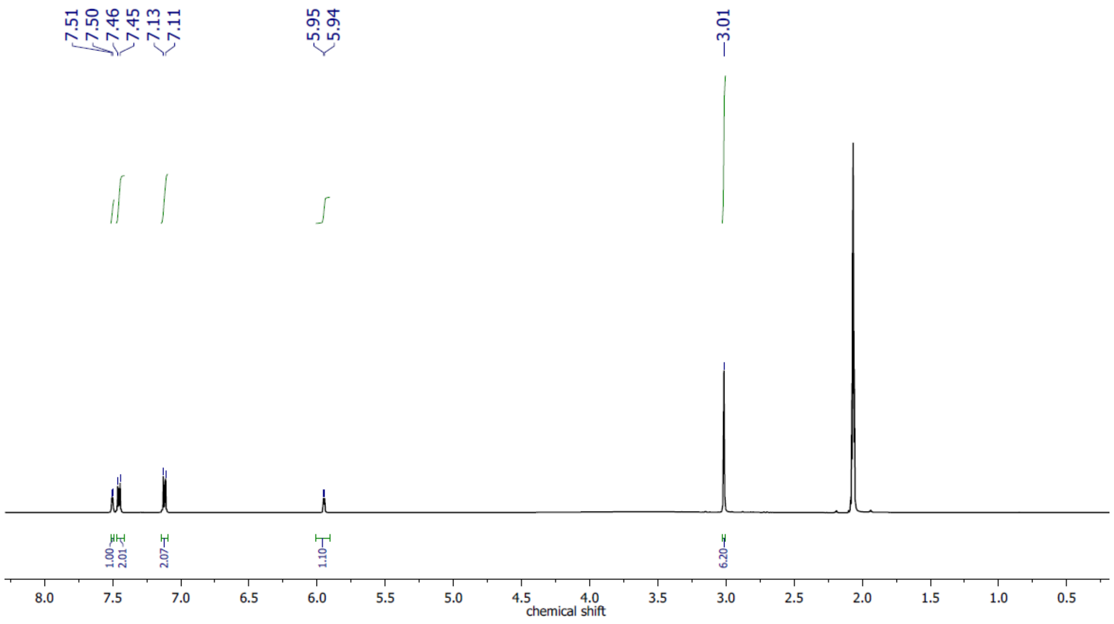

Fig. S12. ^{1}H NMR Spectrum of **IIIi** in $(CD_3)_2CO$.

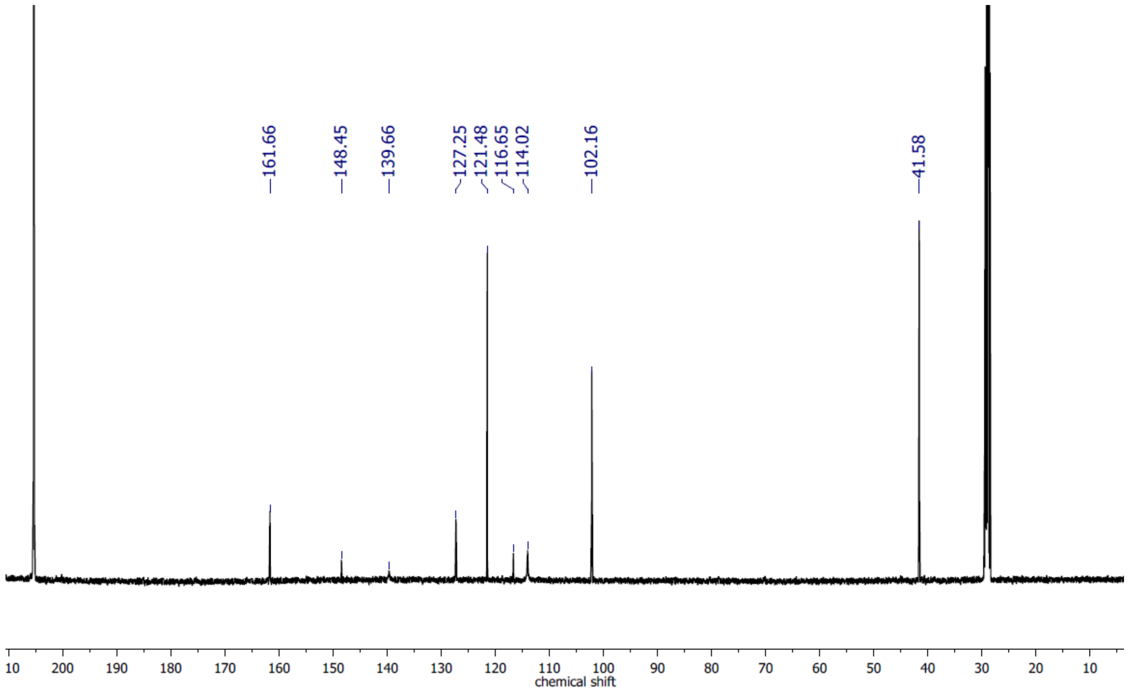

Fig. S13. $^{13}C\{^{1}H\}$ NMR Spectrum of **IIIi** in $(CD_3)_2CO$.

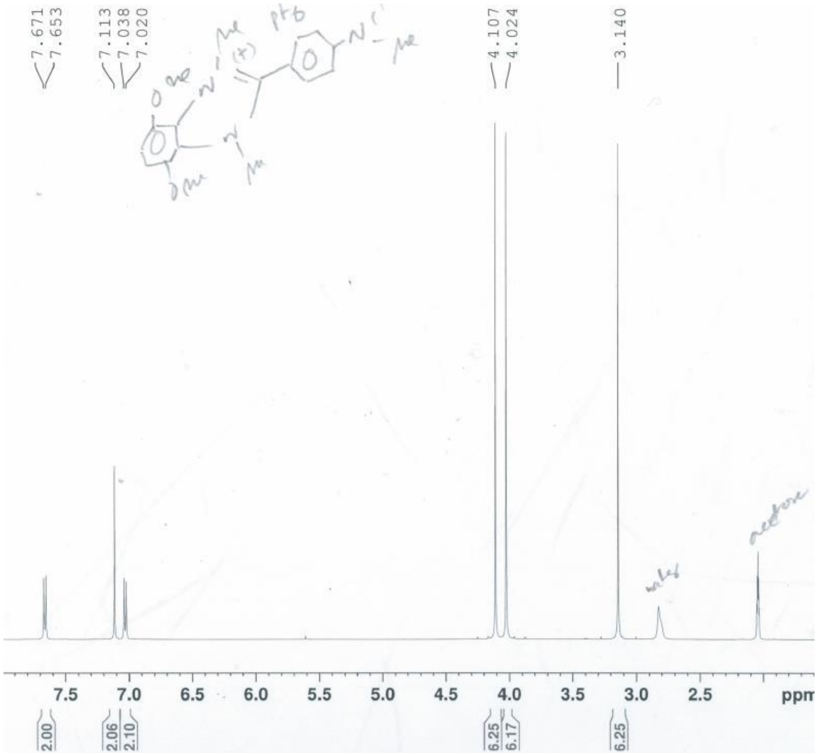

Fig. S14. ^{1}H NMR Spectrum of **1g**$^{+}PF_6^{-}$ in $(CD_3)_2CO$.

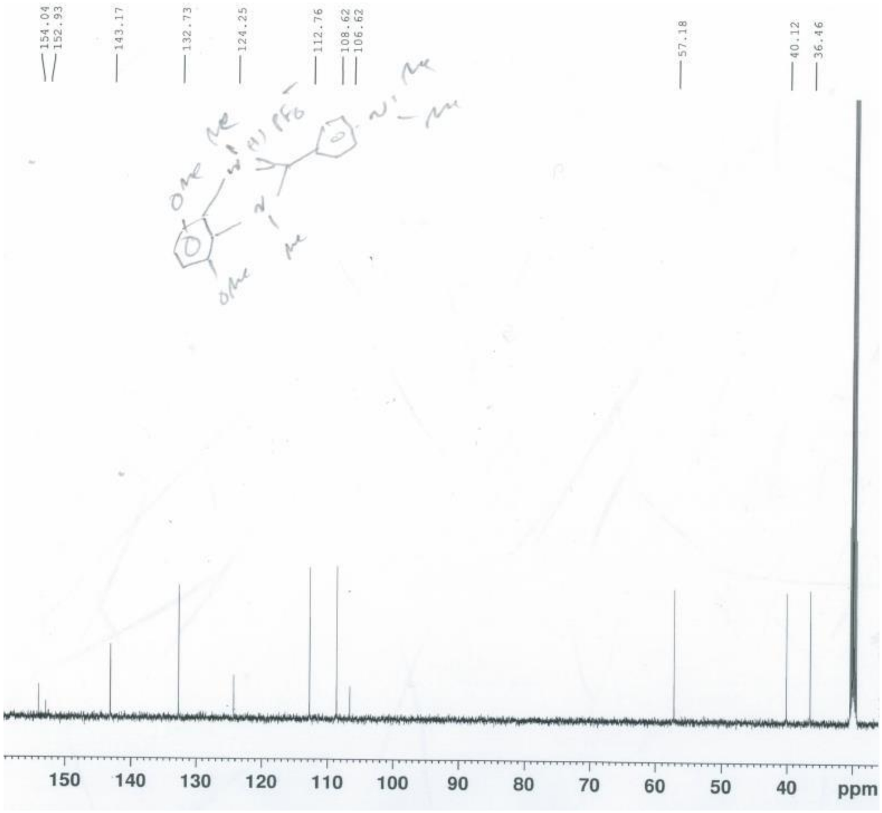

Fig. S15. $^{13}C\{^{1}H\}$ NMR Spectrum of **1g**$^{+}PF_6^{-}$ in $(CD_3)_2CO$.

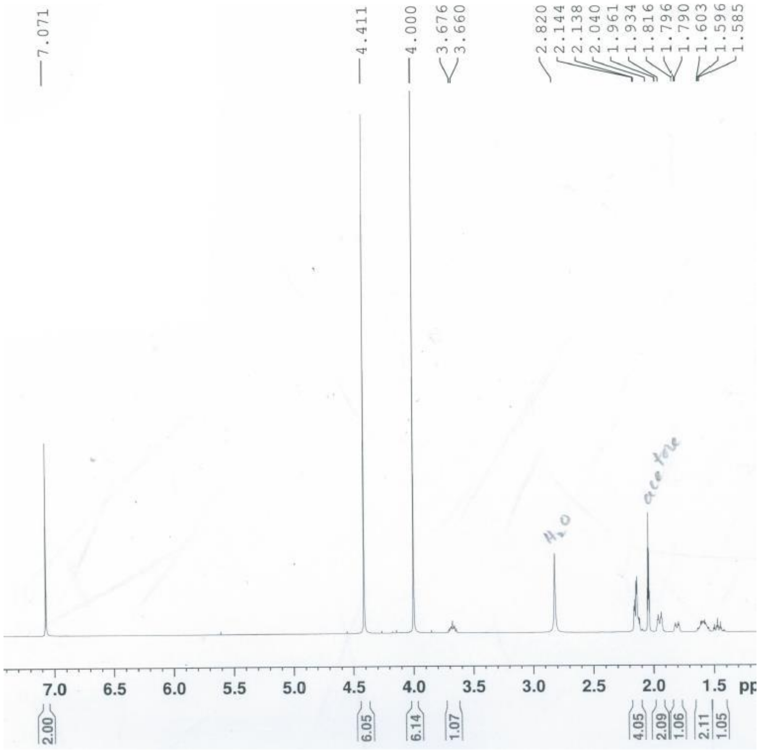

Fig. S16. ^{1}H NMR Spectrum of **1h**$^{+}PF_6^{-}$ in $(CD_3)_2CO$.

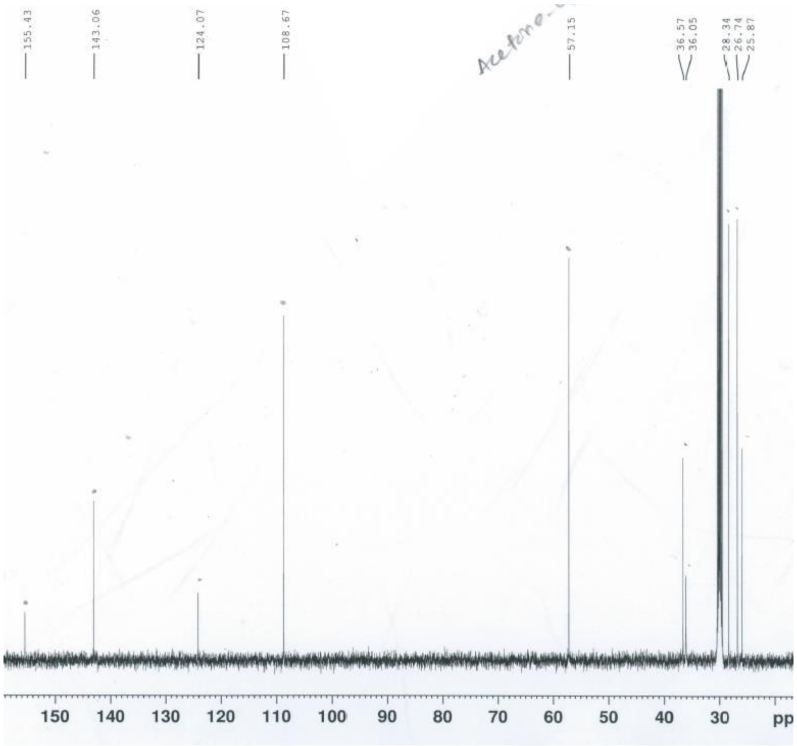

Fig. S17. $^{13}C\{^{1}H\}$ NMR Spectrum of **1h**$^{+}PF_6^{-}$ in $(CD_3)_2CO$.

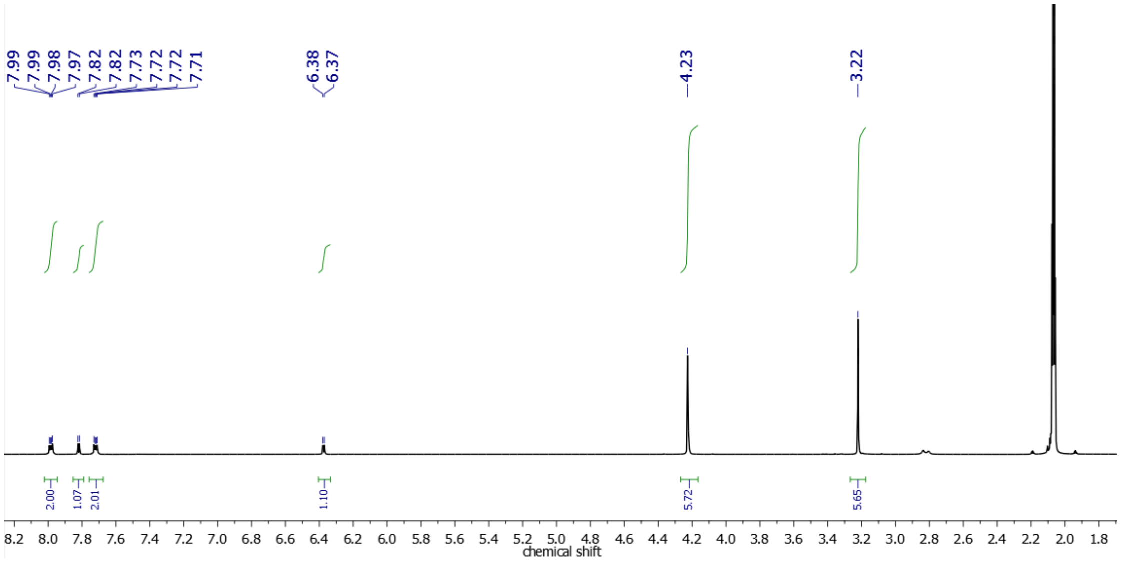

Fig. S18. ^{1}H NMR Spectrum of **1i**$^{+}$$PF_6^{-}$ in $(CD_3)_2CO$.

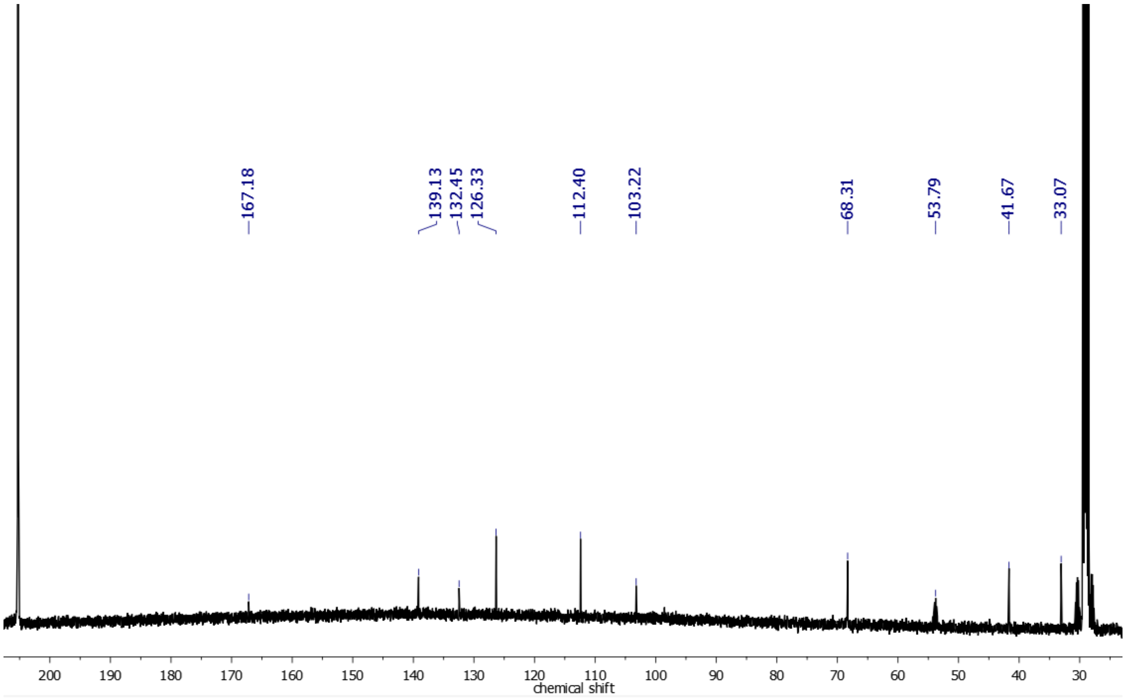

Fig. S19. ^{13}C{^{1}H} NMR Spectrum of **1i**$^{+}$$PF_6^{-}$ in $(CD_3)_2CO$.

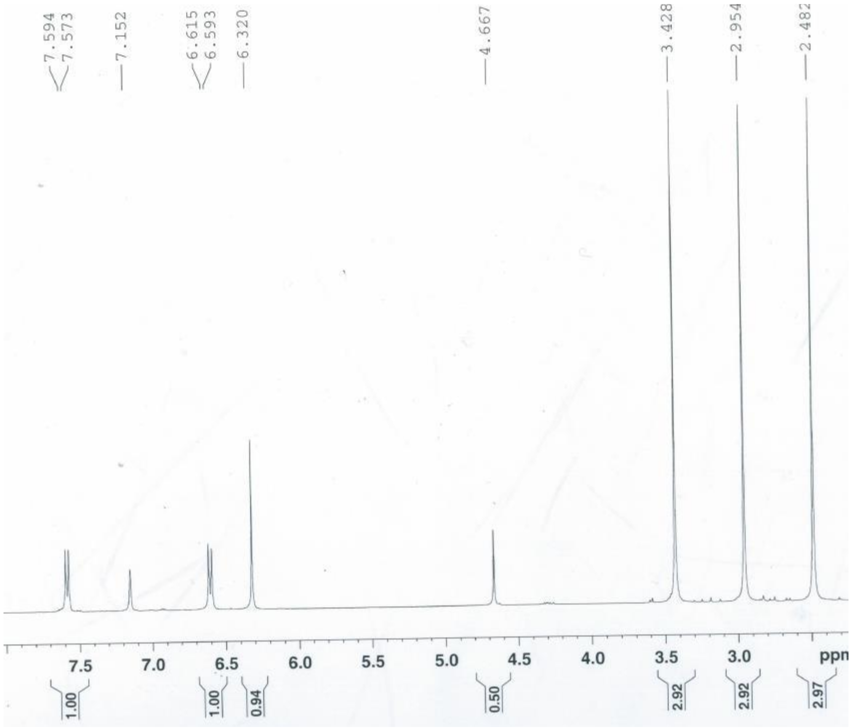

Fig. S20. ^{1}H NMR Spectrum of **1gH** in C_6D_6.

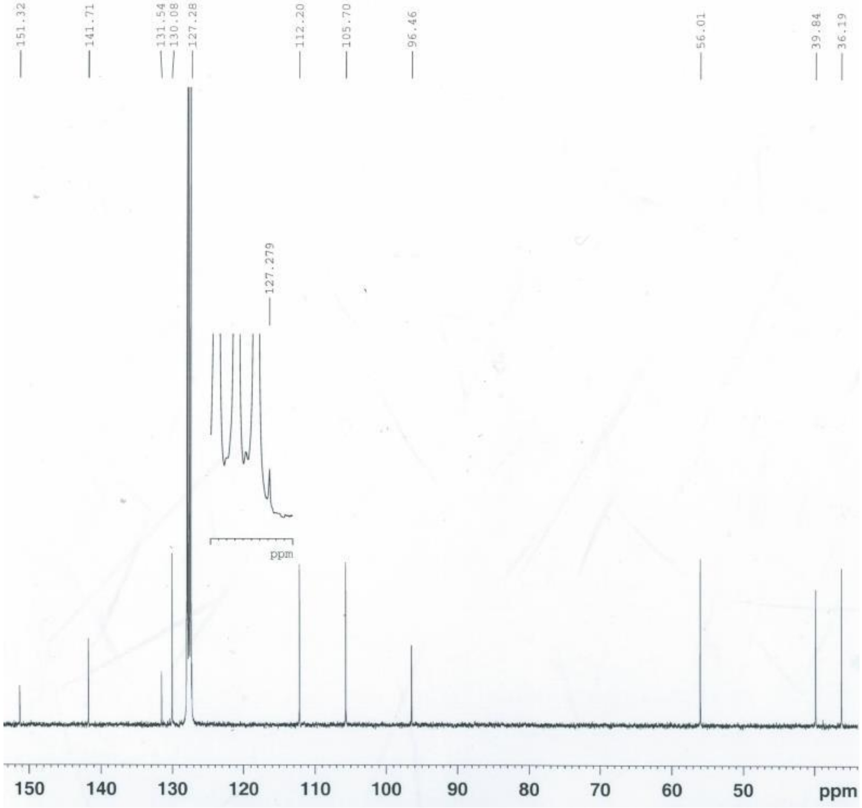

Fig. S21. $^{1}H\{^{13}C\}$ NMR Spectrum of **1gH** in C_6D_6.

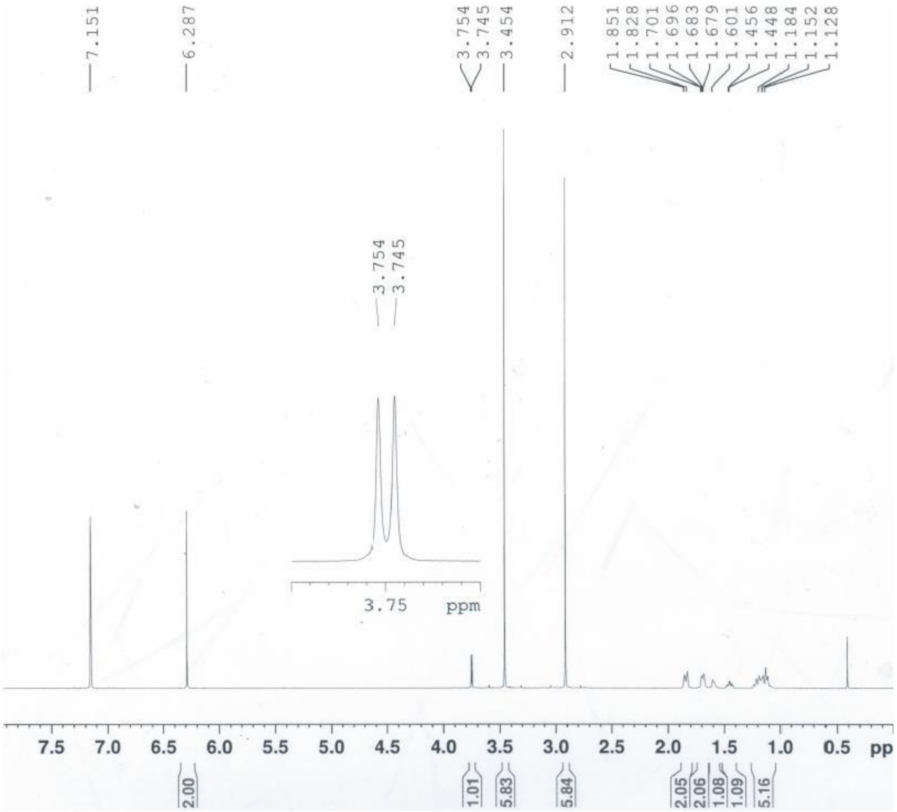

Fig. S22. ^{1}H NMR Spectrum of **1hH** in C_6D_6.

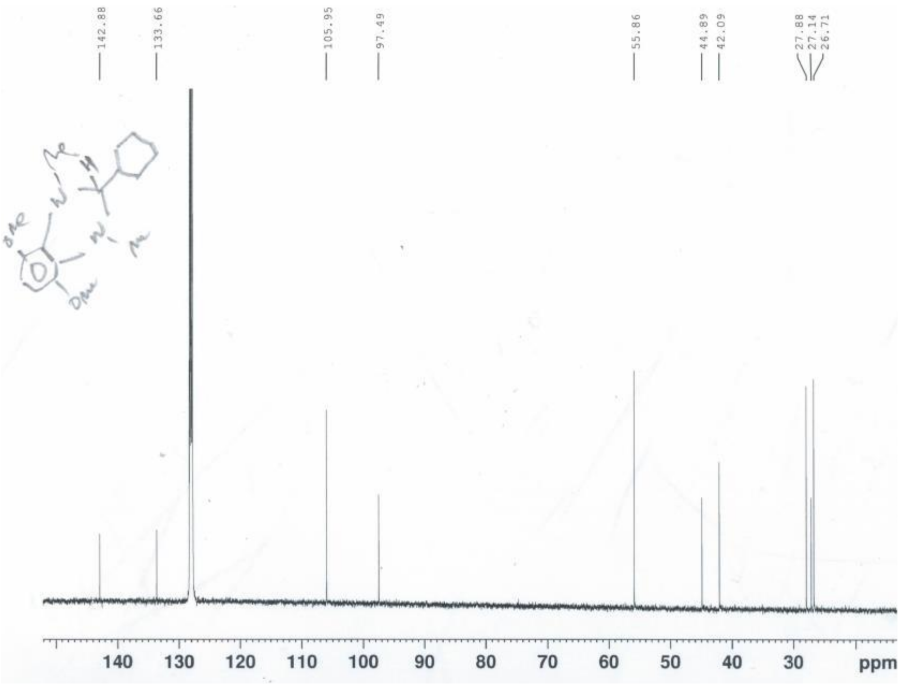

Fig. 23. ^{13}C{^{1}H} NMR Spectrum of **1hH** in C_6D_6.

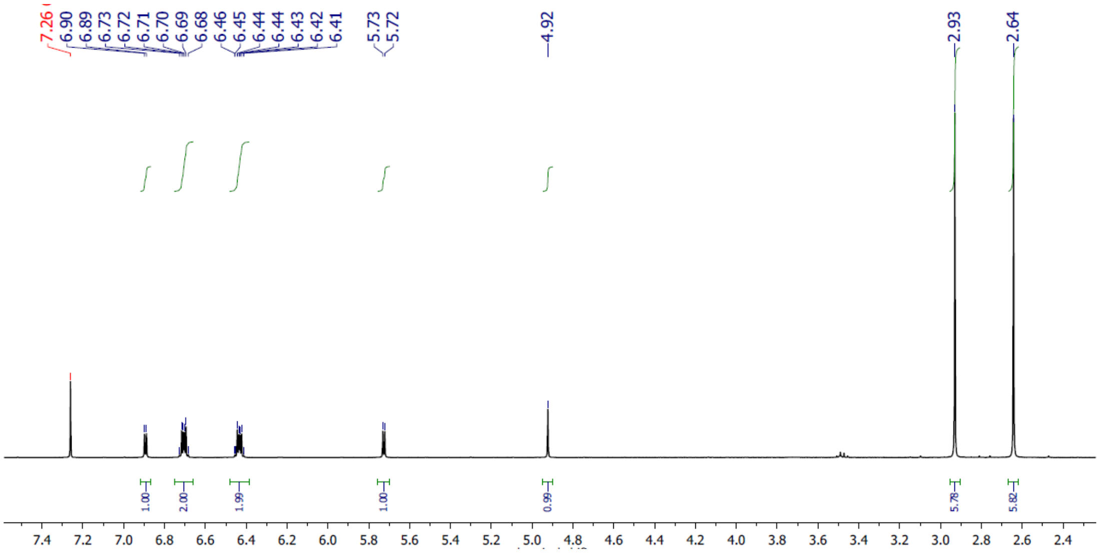

Fig. S24. ^{1}H NMR Spectrum of **1iH** in $CDCl_3$.

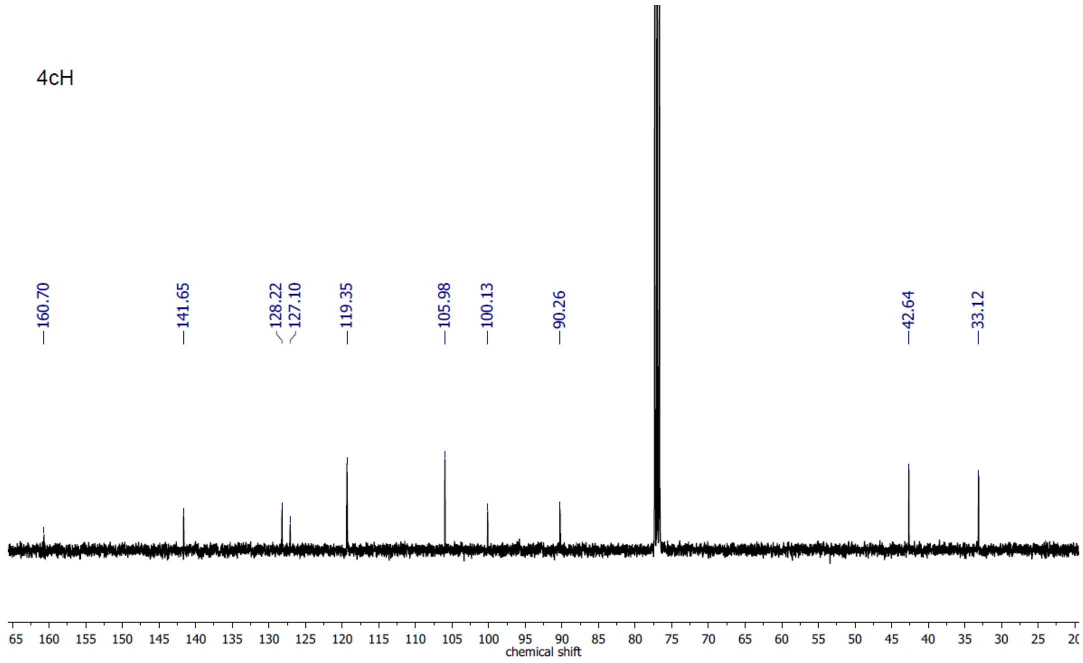

Fig. S25. $^{13}C\{^{1}H\}$ NMR Spectrum of **1iH** in $CDCl_3$.

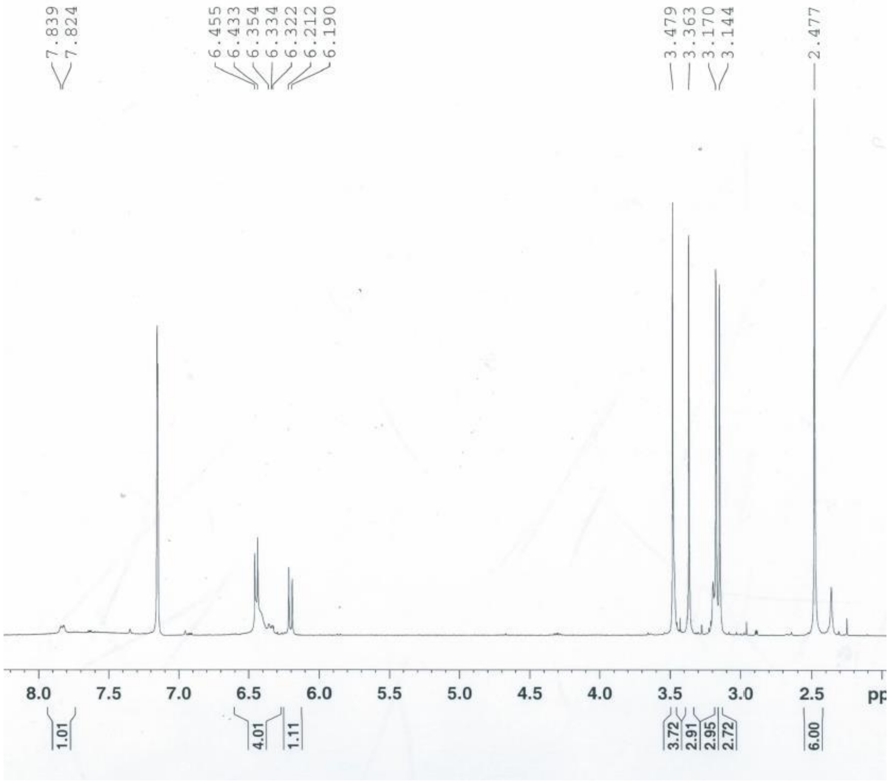

Fig. S26. ^{1}H NMR Spectrum of **1g$_2$** in C_6D_6.

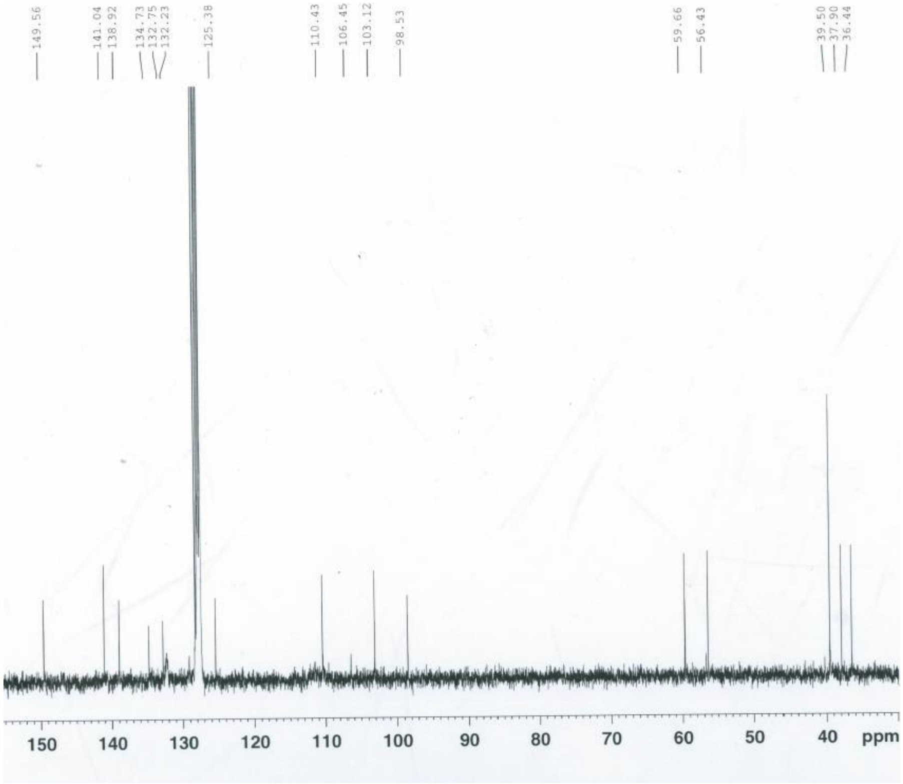

Fig. S27. $^{13}C\{^{1}H\}$ NMR Spectrum of **1g$_2$** in C_6D_6.

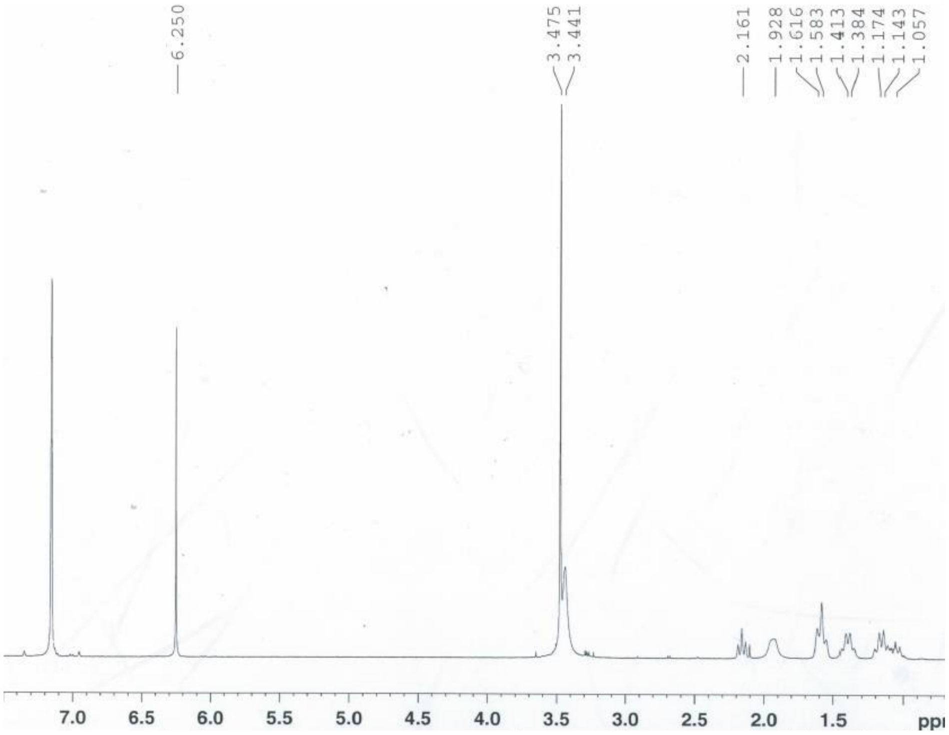

Fig. S28. ^{1}H NMR Spectrum of **1h$_2$** in C_6D_6.

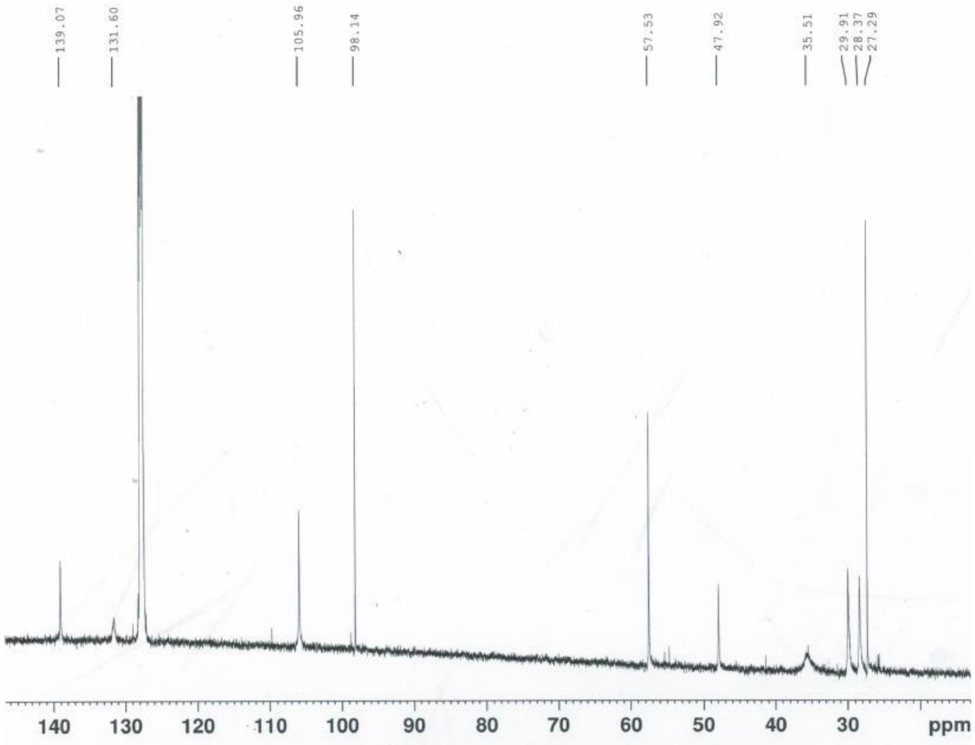

Fig. S29. $^{13}C\{^{1}H\}$ NMR Spectrum of **1h$_2$** in C_6D_6.

7. REFERENCES FOR SUPPORTING INFORMATION

1. Hammershøj, P.; Reenberg, T. K.; Pittelkow, M.; Nielsen, C. B.; Hammerich, O.; Christensen, J. B. *Eur. J. Org. Chem.* **2006**, 2786–2794. doi: 10.1002/ejoc.200600081
2. Un, H.-I.; Gregory, S. A.; Mohapatra, S. K.; Xiong, M.; Longhi, E.; Lu, Y.; Rigin, S.; Jhulki, S.; Yang, C.-Y.; Timofeeva, T. V.; Wang, J.-Y.; Yee, S. K.; Barlow, S.; Marder, S. R.; Pei, J. *Adv. Energy Mater.* **2019**, *9*, 1900817. doi: 10.1002/aenm.201900817
3. Naab, B. D.; Zhang, S.; Vandewal, K.; Salleo, A.; Barlow, S.; Marder, S.; Bao, Z. *Adv. Mater.* **2014**, *26*, 4268–4272. doi: 10.1002/adma.201400668
4. Ghosh, R.; Kushwaha, A.; Das, D. *J. Phys. Chem. B* **2017**, *121*, 8786-8794. doi: 10.1021/acs.jpcb.7b05947
5. Ryu, C.-K.; Ra-YoungLee; Seung-YonLee; Chung, H.-J.; Lee, S. K.; Chung, K.-H. *Bioorg. Med. Chem. Lett.* **2008**, *18*, 2948-2951. doi: 10.1016/j.bmcl.2008.03.066
6. Moriarty, E.; Carr, M.; Bonham, S.; Carty, M. P.; Aldabbagh, F. *Eur. J. Med. Chem* **2010**, *45*, 3762-3769. doi: 10.1016/j.ejmech.2010.05.025
7. Zhu, X.-Q.; Zhang, M.-T.; Yu, A.; Wang, C.-H.; Cheng, J.-P. *J. Am. Chem. Soc.* **2008**, *130*, 2501-2516. doi: 10.1021/ja075523m
8. Naab, B. D.; Guo, S.; Olthof, S.; Evans, E. G. B.; Wei, P.; Millhauser, G. L.; Kahn, A.; Barlow, S.; Marder, S. R.; Bao, Z. *J. Am. Chem. Soc.* **2013**, *135*, 15018–15025. doi: 10.1021/ja403906d
9. Pallini, F.; Mattiello, S.; Cassinelli, M.; Rossi, P.; Mecca, S.; Tan, W. L.; Sassi, M.; Lanzani, G.; McNeill, C. R.; Caironi, M.; Beverina, L. *ACS Appl. Energy Mater.* **2022**, *5*, 2421-2429. doi: 10.1021/acsaem.1c03893
10. Zhang, S.; Naab, B. D.; Jucov, E. V.; Parkin, S.; Evans, E. G. B.; Millhauser, G. L.; Timofeeva, T. V.; Risko, C.; Brédas, J.-L.; Bao, Z.; Barlow, S.; Marder, S. R. *Chem. Eur. J.* **2015**, *21*, 10878-10885. doi: 10.1002/chem.201500611
11. Zhang, S.; Moudgil, K.; Jucov, E.; Risko, C.; Timofeeva, T. V.; Marder, S. R.; Barlow, S. *Inorg. Chim. Acta* **2019**, *489*, 67-77. doi: 10.1016/j.ica.2019.02.003
